# Reference Assembly and Annotation of the *Pyrenophora teres* f. *teres* Isolate 0-1

**DOI:** 10.1534/g3.117.300196

**Published:** 2017-11-21

**Authors:** Nathan A. Wyatt, Jonathan K. Richards, Robert S. Brueggeman, Timothy L. Friesen

**Affiliations:** * Genomics and Bioinformatics Program, North Dakota State University, Fargo, North Dakota 58102; † Department of Plant Pathology, North Dakota State University, Fargo, North Dakota 58102; ‡ Cereal Crops Research Unit, Red River Valley Agricultural Research Center, United States Department of Agriculture-Agricultural Research Service (USDA-ARS), Fargo, North Dakota 58102

**Keywords:** *Pyrenophora teres* f. *teres*, genome sequencing, RNAseq, PacBio, barley, genome report

## Abstract

*Pyrenophora teres* f. *teres*, the causal agent of net form net blotch (NFNB) of barley, is a destructive pathogen in barley-growing regions throughout the world. Typical yield losses due to NFNB range from 10 to 40%; however, complete loss has been observed on highly susceptible barley lines where environmental conditions favor the pathogen. Currently, genomic resources for this economically important pathogen are limited to a fragmented draft genome assembly and annotation, with limited RNA support of the *P. teres* f. *teres* isolate 0-1. This research presents an updated 0-1 reference assembly facilitated by long-read sequencing and scaffolding with the assistance of genetic linkage maps. Additionally, genome annotation was mediated by RNAseq analysis using three infection time points and a pure culture sample, resulting in 11,541 high-confidence gene models. The 0-1 genome assembly and annotation presented here now contains the majority of the repetitive content of the genome. Analysis of the 0-1 genome revealed classic characteristics of a “two-speed” genome, being compartmentalized into GC-equilibrated and AT-rich compartments. The assembly of repetitive AT-rich regions will be important for future investigation of genes known as effectors, which often reside in close proximity to repetitive regions. These effectors are responsible for manipulation of the host defense during infection. This updated *P. teres* f. *teres* isolate 0-1 reference genome assembly and annotation provides a robust resource for the examination of the barley–*P. teres* f. *teres* host–pathogen coevolution.

Net form net blotch (NFNB) of barley (*Hordeum vulgare*) is caused by the fungal pathogen *Pyrenophora teres* f. *teres*. Globally, NFNB results in regular yield losses of between 10 and 40% with the potential for complete losses in environmental settings favorable to the pathogen, namely, susceptible cultivars with high sustained humidity and the absence of fungicides (Mathre *et al.* 1997; Liu *et al.* 2011). Several studies have investigated the genetics of this host–pathogen interaction, utilizing biparental mapping populations of both the host and pathogen as well as genome-wide association studies (GWAS) in the host (Liu *et al.* 2011; Shjerve *et al.* 2014; Carlsen *et al.* 2017; Koladia *et al.* 2017a; Richards *et al.* 2017). These studies have been critical in developing hypothetical models for this pathosystem. These models have proposed that the *P. teres* f. *teres*-barley interaction involves the production of effectors that are involved in manipulating the host to gain an advantage (Koladia *et al.* 2017a), and that some of these effectors may be recognized by dominant resistance genes (Koladia *et al.* 2017b), showing hallmarks of an effector triggered susceptibility/effector triggered immunity type model as described by Chisholm *et al.* (2006) and Jones and Dangl (2006). *P. teres* f. *teres* has also been shown to produces necrotrophic effectors (NE) that are involved in NE-triggered susceptibility (Liu *et al.* 2015; Shjerve *et al.* 2014) when recognized by dominant host susceptibility genes (Abu Qamar *et al.* 2008; Liu *et al.* 2010). Together, these studies indicate a complex interaction where selection pressure has been placed on the pathogen to produce different types of effectors to manipulate its host.

Currently, 1084 fungal genomes have been sequenced and deposited at the National Center for Biotechnology Information (NCBI) (as of August 2017, source: http://www.ncbi.nlm.nih.gov/genome/browse/), and many of these genomes remain fragmented. The genomic regions responsible for this fragmentation are typically repetitive regions, which are common among fungal “two-speed” genomes, a term used for genomes that have a distinct bipartite architecture made up of typical gene-rich regions and repeat-rich, gene-poor regions thought to be hotbeds for genome evolution (Dong *et al.* 2015). Genome assemblies that rely on short-read technologies struggle to span these repetitive regions as the repeats are often collapsed (Ekblom and Wolf 2014). Effector genes involved in the manipulation of the plant defenses cluster proximal to, and within, these repeat regions (Thomma *et al.* 2016), and, therefore, it is critical that these regions be accounted for in any genome assembly of a plant pathogen being used for effector discovery.

The current reference isolate of *P. teres* f. *teres* is the Canadian isolate 0-1. The original draft genome sequence of 0-1 was reported in 2010, and used paired-end Illumina sequencing at roughly 20× coverage, resulting in an assembly with a total size of 41.95 Mb (Ellwood *et al.* 2010). This assembly contained 11,799 gene models, providing useful tools for the interrogation of the genome; however, the genome still remained fragmented at 6412 contigs with an N50 of only 30,037 bp. The fragmented nature of this assembly presented obstacles to the study of the genome of *P. teres* f. *teres*, especially in regards to map based cloning using biparental mapping populations.

New long-read sequencing technologies [*e.g.*, Pacific Biosciences (PacBio) single molecule real-time (SMRT) sequencing platform] are capable of producing reads up to 60 kb (Goodwin *et al.* 2016), and, currently, two fungal plant pathogens have been fully sequenced using these long read technologies (Faino *et al.* 2015; Van Kan *et al.* 2017). These results demonstrate the utility of long-read technologies in the production of better assemblies of fungal plant pathogens.

Here, we present an updated, reference quality genome assembly and annotation for the *P. teres* f. *teres* isolate 0-1. In order to update the reference assembly and annotation, we sequenced a total of 14 SMRT cells at the National Center for Genome Resources (NCGR), and conducted RNAseq using both *in vitro* and *in planta* samples. The updated 0-1 assembly is currently in 86 contigs, with a total genomic content of 46.5 Mb, with a high-confidence annotation set of 11,541 gene models. This updated assembly and annotation presents a useful tool for the genomic interrogation of *P. teres* f. *teres*, and, specifically, the investigation of the secretome and effectorome.

## Materials and Methods

### Biological materials and high-molecular-weight DNA extraction

*P. teres* f. *teres* isolate 0-1 is a Canadian isolate collected from Ontario (Weiland *et al.* 1999). Fungal tissue for DNA extraction was obtained in a manner similar to that reported by Shjerve *et al.* (2014). Briefly, a single dried fungal plug was placed on a V8-PDA plate and allowed to grow for 5 d, after which the culture underwent a 24-hr light and dark cycle to induce sporulation. Spores were then inoculated into Fries medium [5 g (NH_4_)_2_C_4_H_4_O_6_, 1 g NH_4_NO_3_, 0.5 g MgSO_4_•7H_2_O, 1.3 g KH_2_PO_4_, 5.48 g K_2_HPO_4_•3H_2_O, 30 g sucrose, 1 g yeast extract, 2 ml trace element stock solution (167 mg LiCl, 107 mg CuCl•H_2_O, 34 mg H_2_MoO_4_, 72 mg MnCl_2_•4H_2_O, 80 mg CoCl_2_•4H_2_O, ddH_2_O to 1 liter)] and allowed to grow for 5 d. Five-day cultures were blended and inoculated into new Fries medium, and allowed to grow for another 24 hr before harvesting.

Harvested tissue was ground to a fine powder under liquid nitrogen with a mortar and pestle, and then placed in a 50 ml conical tube. Next, 25 ml of Qiagen RLT buffer and 150 µl RNAse A at 20 mg/ml was added to the 50 ml conical tube containing fungal tissue. The mixture was homogenized by pipetting and vortexing until well mixed, and incubated at 65° for 45 min, mixing at 15-min intervals. The 50 ml conical tube was centrifuged at 3166 × *g* for 15 min and the resulting supernatant was split between two Oakridge tubes. Equal volumes of 25:24:1 phenol:chloroform:isoamyl alcohol was added to each tube and mixed gently and thoroughly by rocking. After mixing, tubes were centrifuged in a fixed angle rotor for 20 min at 13,000 × *g* at room temperature. The aqueous layer was drawn off, so as not to disturb the middle phase, and placed in a new 50 ml conical tube; 0.4 volumes of sodium acetate and an equal volume of isopropyl alcohol were added to the 50 ml tube, and the solution was mixed and incubated at room temperature for 30 min to precipitate the DNA. DNA was removed from the 50 ml conical tube using a glass hook and placed in a clean weigh boat and subsequently rinsed twice using 2–5 ml of freshly prepared 70% ethanol. Ethanol was then pulled off via pipetting and the DNA was moved to a 5 ml tube and placed in a lyophilizer for 30 min to dry. DNA was rehydrated with 1 ml of molecular biology grade water, and incubated at 4° overnight. DNA was quantified using a Qubit (ThermoFischer Scientific), and sample concentration was adjusted to 1 µg/ml.

### Genomic sequencing and de novo assembly

Genomic DNA was shipped on dry ice to NCGR (Santa Fe, NM) for library preparation and sequencing. Whole-genome shotgun sequencing was performed at NCGR using the PacBio RSII instrument with a 20 kb size selected library and current P6-C4 chemistry. A total of 14 SMRT cells was sequenced for *P. teres* f. *teres* isolate 0-1.

Raw reads in the form of FASTQ files were input into the Canu assembler (Koren *et al.* 2017) under default parameters for correction, trimming, and assembly with a genome size estimate of 42 Mb. A second iteration of genome assembly was then done with the same parameters, but a larger genome estimate of 46.5 Mb based on the first draft assembly. Pilon v1.21 (Walker *et al.* 2014) was used to polish the 0-1 assembly to improve local base calling accuracy. Pilon takes the reference assembly FASTA file and a BAM file of the aligned reads as an input to identify miscalled bases, small indels, or large structural misassemblies for correction, and outputs a new FASTA file containing the polished genome assembly.

### Genetic mapping and genome scaffolding

A genetic linkage map was created from the biparental population 0–1 × 15A consisting of 120 progeny isolates, 78 isolates were obtained from Lai *et al.* (2007), and an additional 42 isolates were obtained from another 0–1 × 15A cross following the methods described in Koladia *et al.* (2017a). Single nucleotide polymorphic (SNP) markers were identified following a RAD-GBS pipeline also described in Koladia *et al.* (2017a). A total of 10 progeny isolates were dropped from the analysis due to large amounts of missing data (>75%), and five isolates were dropped after being identified as parental clones, bringing the total to 105 progeny isolates. Linkage mapping was conducted in MapDisto v1.7.9 (Lorieux 2012) as described in Koladia *et al.* (2017a) using a LOD threshold of 5.0.

*P. teres* f. *teres* isolate 0-1 assembled contigs were scaffolded using ALLMAPS (Tang *et al.* 2015). ALLMAPS takes assembled contigs and generates scaffolds based on coordinates from genetic linkage maps, optical maps, or syntenic maps. To scaffold the 0-1 assembly, linkage maps from the biparental populations 0–1 × 15A and the recently published FGOH04-Ptt21 × BB25 population (Koladia *et al.* 2017a) were input into ALLMAPS and merged to a single coordinate BED file. The merged coordinate BED file was input with the 0-1 genome FASTA file for scaffolding under default parameters within ALLMAPS.

### RNA sequencing and assembly

Cultures and inoculum of *P. teres* f. *teres* isolate 0-1 were prepared as previously described (Koladia *et al.* 2017a). Seeds of barley line “Tifang” were sown into a 96-conetainer rack with a border of Tradition barley and grown under greenhouse conditions for ∼2 wk. For each RNAseq sample time point, five individual containers containing two seedlings each were selected for inoculation. The second fully extended leaf of each seedling was taped flat to a 24 × 30 cm plastic surface so as to provide a flat surface to evenly coat sample leaves with inoculum. Following inoculation, plants were placed into a lighted mist chamber for 24 hr with 100% relative humidity. After 24 hr, plants were moved to a growth chamber with a temperature of 21° and a 12-hr photoperiod. Samples were collected by punching circular leaf discs from predesignated regions of the leaf using a sterile hole punch. Each leaf was punched a total of five times equating to 50 tissue samples per collected time point. Tissue was immediately flash frozen in liquid nitrogen and stored at −80° until RNA extraction. Liquid cultures of isolate 0-1 were prepared by incubating collected fungal spores in 75 ml of Fries medium (Koladia *et al.* 2017a) for 5 d. Tissue was harvested, rinsed, flash frozen in liquid nitrogen, and stored at −80° until RNA extraction. RNA sequencing was done using *in planta* time points of 48, 72, and 96 hr postinoculation, and a sample from pure culture. Both the pure culture and *in planta* samples were collected in three replicates. mRNA from each sample was extracted using the mRNA Direct Kit (ThermoFisher Scientific) following the manufacturer’s protocol. RNAseq library preparation was done with the Illumina Truseq v.3 kit following the manufacturer’s protocol. Quality and fragment size distribution of the prepared libraries were examined using an Agilent DNA chip on a bioanalyzer (Agilent, Santa Clara, CA). Libraries were sequenced on an Illumina Nextseq at the USDA-ARS Small Grains Genotyping Center (Fargo, ND) to produce 150 bp single-end reads.

The output of the sequencing run was parsed with the open source program bcl2fastq2 (Illumina) and reads were input to FastQC for quality inspection (Andrews 2011). Trimmomatic was used to trim raw reads using the parameters HEADCROP:15, ILLUMINACLIP:2:30:10 with Illumina adapter and index sequences provided, and SLIDINGWINDOW:4:15 (Bolger *et al.* 2014). Trimmed reads were aligned to the 0-1 genome sequence using HISAT, and aligned reads were assembled and analyzed using StringTie following the protocol laid out in Pertea *et al.* (2016). Briefly, reads were aligned to the genome using HISATv2 with the option “max-intronlen=3000,” which is suggested for fungal genomes (Ter-Hovhannisyan *et al.* 2008). Aligned reads were output in SAM format, and converted to sorted BAM files. These BAM files were input into StringTie for assembly of transcripts (Pertea *et al.* 2016).

### Genome annotation

Gene models were determined using the Maker2 pipeline (Holt and Yandell 2011). The Maker2 pipeline incorporates multiple sources of evidence, and leverages these to create the most accurate gene models possible. *Ab initio* annotations were provided through Augustus with the model organisms *Neurospora crassa* training set (Stanke and Morgenstern 2005) and Genemark-ES v.2 (Ter-Hovhannisyan *et al.* 2008), which contains a self-training algorithm. Assembled RNAseq transcripts were also provided to the pipeline as a GFF3 file along with external protein evidence from the closely related species *Pyrenophora tritici-repentis* (Manning *et al.* 2013) and the current NCBI *P. teres* f. *teres* 0-1 annotation (Ellwood *et al.* 2010) in fasta format. Options within the Maker2 pipeline that were used in the first iteration of annotation were “est2genome=1” and “protein2genome=1,” which instruct the pipeline to use evidence from RNAseq data and BLAST results of supplied proteins to build gene models. These gene models were then used to train the *ab initio* annotation program SNAP (Korf 2004), and the pipeline was rerun with the addition of a SNAP training file specific to the 0-1 genome. The output from this second Maker2 pipeline run was then used to retrain SNAP a second time, and subsequently rerun to further refine gene models (Holt and Yandell 2011). To evaluate the quality of the updated 0-1 assembly gene models, RNAseq transcript coverage was calculated using BEDtools “coverage” (Quinlan and Hall 2010) with Maker2 gene models and aligned transcripts input in BED format. RNAseq evidence for a gene model was defined as having >50% transcript coverage of a gene model.

To evaluate the completeness of the assembly’s annotated gene models, the program BUSCO was applied to the updated 0-1 genome assembly and annotation. BUSCO utilizes sets of core genes in taxon-specific databases to evaluate the relative completeness of the assembly and annotation. For the purposes of assessing the 0-1 genome, the BUSCO database for Ascomycota was used, as it was the most specific data set in the BUSCO databases relating to *P. teres* f. *teres*. The Ascomycota data set contains 1315 genes curated from 75 different Ascomycota species (Simão *et al.* 2015). To compare the completeness of the 0-1 updated assembly, three other closely related species were downloaded from NCBI and evaluated with BUSCO; *P. tritici repentis Pt-1C-BFP* (ASM14998v1), *Parastagonospora nodorum SN15* (ASM14691v2), and *Leptosphaeria maculans JN3* (ASM23037v1).

RepeatModeler v1.0.11 (Smit *et al.* 2013–2015) was used to *de novo* annotate repetitive elements in the genome in order to create a custom *P. teres* f. *teres* repeat library. The RepeatModeler *P. teres* f. *teres* repeat library was input into RepeatMasker (Smit and Hubley 2008-2015 alongside the current release of Repbase (v22.10) (Bao *et al.* 2015), to soft mask identified repetitive elements and output a final annotation of repetitive elements identified in the newly assembled 0-1 *P. teres* f. *teres* genome. The “buildSummary.pl” RepeatMasker script was applied to gather summary statistics for downstream analysis of repetitive elements.

### Secretome, effectorome, GC content structure, and repetitive analysis

Secreted proteins were identified using SignalP v4 (Petersen *et al.* 2011) and output as mature proteins, lacking the signal sequence. Mature secreted proteins were input into EffectorP v1 (Sperschneider *et al.* 2016) to identify putative effectors in the updated 0-1 annotation set.

OcculterCut v1 (Testa *et al.* 2016) is a tool used to identify GC content patterns in genomes. OcculterCut outputs a number of useful statistics, which include the average sizes of GC-rich and poor regions, as well as average gene densities within each region. OcculterCut was run with the updated 0-1 genome input as a FASTA file, the updated 0-1 annotation in GFF3 format, and default run parameters. RIPCAL (Hane and Oliver 2008) was implemented to scan for evidence of repeat-induced point mutations (RIP) within the repetitive content of the 0-1 genome. Repeat family sequences of >400 bp of the five most common repeat families were subjected to RIPCAL to determine RIP dominance. The TpA/ApT RIP index and the (CpA+TpG)/(ApC+GpT) RIP index were additionally computed and compared to the RIP indices of a randomly parsed set of sequences from the 0-1 genome. Random sequences of similar size were parsed using the custom Perl script supplied in Derbyshire *et al.* (2017) for a total of 50 sequences.

### Whole-genome alignment

Contigs from the first assembly of *P. teres* f. *teres* isolate 0-1 (Ellwood *et al.* 2010) were aligned to the newly assembled reference with the alignment program “nucmer” within the MUMmer v3.0 package (Delcher *et al.* 2003) using the “mum” option to compute maximal unique matches in the references and query sequences. Alignments were converted to a BED file for downstream analysis. Genome coverage for the 12 reference 0-1 scaffolds were calculated using Bedtools v2.26.0 “coverage” (Quinlan and Hall 2010) to output a percent coverage of each scaffold.

Genomic windows consisting of 1000 bp of the reference 0-1 assembly were calculated using Bedtools “makewindows” (Quinlan and Hall 2010) in order to compare coverage statistics relative to repeat regions of the genome. Genomic regions containing low- to no-coverage were identified as having <200 bp of overlap within a 1000 bp genomic region. These low- to no-coverage regions were then compared to regions of the genome harboring repetitive element using Bedtools v2.26.0 “coverage” (Quinlan and Hall 2010). Genomic regions containing low- to no-coverage, and also having 50% of the region covered by a repetitive element, were output.

### Data availability

Sequence and annotation data are available at NCBI GenBank under BioProject PRJNA392275. Figure S1 in File S1 contains figures representing marker positions along scaffolded *P. teres* f. *teres* isolate 0-1 assembly contigs. Figure S2 in File S1 contains RIPCAL outputs of RIP dominance observed in the five most numerous repeat families annotated in the 0-1 assembly. Table S1 in File S1 contains summary data of RepeatModeler annotations. Table S2 in File S1 contains calculated RIP indices of the five move numerous repeat families and a randomly parsed set of genomic sequences.

## Results and Discussion

### Sequencing and de novo genome assembly

PacBio SMRT sequencing of *P. teres* f. *teres* isolate 0-1 generated a total of 1,148,507 reads from the 14 SMRT cells sequenced, with an average read length of 8051 bp. A total of 9,246,774,161 bp were obtained, equating to ∼200× coverage of the 0-1 genome.

Assembling this data using the Canu assembler yielded a fairly contiguous assembly, with 85 total contigs and one contig representing the mitochondrial genome (86 total contigs). The total size of the assembly was ∼46.5 Mb, with an N50 of 1,730,401 bp, and an L50 of 11 contigs. This assembly provides a drastic improvement from the previous assembly based on the quality metrics summarized in Table 1. The use of long-read technology allowed for the assembly of low complexity, repeat dense regions, which are difficult to assemble using short-read technologies. The increased resolution of the genome will aid in the investigation of evolutionarily active regions, which are often repeat-rich and harbor important genes related to pathogen adaptation.

**Table 1:** Updated 0-1 assembly summary statistics compared to previous 0-1 assembly

Feature	Updated 0-1 Assembly	Previous 0-1 Assembly*^a^*
Genome size	46,508,966	41,957,260
Total contigs	86	6,412
Largest contig	3,573,185	300,442
Smallest contig	27,932	200
Prescaffolding L50*^b^*	11	408
Prescaffolding N50*^c^*	1,730,401	26,790
Contigs <100 kb	33	6,389
GC%*^d^*	46	48
Telomeres*^e^*	9	0

a Ellwood *et al.* (2010).

b Smallest number of contigs whose length equals 50% of the prescaffolding genome assembly.

c Length of the smallest contig in an ordered set of contigs corresponding to 50% of the prescaffolding assembly length.

d Overall GC% content of the 0-1 genome assembly.

e Number of telomeres identified in the 0-1 assembly on the end of contigs.

### Genetic mapping and genome scaffolding

Ion Torrent RAD-GBS sequencing generated reads for parental isolates 0-1 and 15A, and the 120 progeny. Reads were aligned to the updated *P. teres* f. *teres* 0-1 genome, and a total of 284 SNP markers was identified. SNPs were input into MapDisto v1.7.9 following a filtering process for genetic mapping. A total of 17 linkage groups (LGs) was obtained, with sizes that ranged from 9.69 to 159.44 cM, and totaling a genetic distance of 987.36 cM.

The 0-1 × 15A genetic map appears to be at low resolution by comparison to the FGOH04 × BB25 genetic map consisting of 16 LGs with 685 SNP markers (370 nonredundant markers) spanning a genetic distance of 1905.81 cM (Koladia *et al.* 2017a). To increase the resolution of the genetic map used for scaffolding the updated 0-1 assembly, the 0-1 × 15A SNPs and the FGOH04 × BB25 SNPs were combined into a single coordinate BED file using ALLMAPS (Tang *et al.* 2015).

Genome scaffolding using ALLMAPS (Tang *et al.* 2015) was accomplished with the combined 0-1 × 15A and FGOH04 × BB25 genetic maps (Koladia *et al.* 2017a) input as a single merged coordinate BED file and the updated 0-1 genome assembly in FASTA format. Scaffolding resulted in 12 scaffolds containing 43 of the 85 contigs in the updated 0-1 assembly, and represents 91.8% of the total base pairs in the 0-1 assembly (Table 2). Marker density across the 12 scaffolds equaled 15.3 markers per Mbp, with 652 of the markers uniquely anchored in the scaffolds (Table 2). Unscaffolded contigs ranged in size from ∼28 to ∼367 kb, and represented 8.2% of the 0-1 genome. ALLMAPS scaffolding statistics are summarized in Figure S1 in File S1 and Table 2 contains graphics depicting marker locations across the scaffolded assembly.

**Table 2:** ALLMAPS genome scaffolding statistics

Feature	Scaffolded 0-1 Assembly
Markers	652
Markers per Mb	15.3
Scaffolded contigs	43
Scaffolded bases	42,704,288
Unscaffolded contigs*^a^*	42
Unscaffolded bases*^b^*	3,804,678
Scaffold N50*^c^*	4,379,536
Scaffold L50*^d^*	5
Genome % scaffolded	91.8%
Total scaffolds	12

a Contigs lacking a marker from either linkage map used when scaffolding.

b Total bases in the unscaffolded contigs.

c Smallest number of scaffolds whose length equals 50% of genome assembly.

d Length of the smallest scaffold in an ordered set of scaffolds corresponding to 50% of the assembly length.

### Genome annotation and assessment

Genome annotation via the Maker2 pipeline yielded 11,541 genes or pseudogenes (Table 3). Evidence for the updated 0-1 gene annotations were derived from either imported protein sequences from the current genome annotation of 0-1 (Ellwood *et al.* 2010), or from the closely related species *P. tritici-repentis* (Manning *et al.* 2013), as well as *ab initio* annotations. RNAseq evidence was present for 72.9% (8414) of the gene annotations. illustrating the high level of confidence for these gene models. Many of the gene models without RNAseq evidence are likely to be involved in the saprotrophic stage of the *P. teres* f. *teres* life cycle due to the samples being collected only from culture and early *in planta* time points.

**Table 3:** Gene annotation summary statistics

Parameter	Value
Genes	11,541
Mean gene length	1,470
Max gene length	43,584
Min gene length	60
Mean exons/gene	3
Predicted secreted proteins*^a^*	1,002
Predicted effectors*^b^*	282

a Proteins harboring predicted signal sequence via SignalP (Petersen *et al.* 2011).

b Secreted proteins predicted to be effectors via EffectorP (Sperschneider *et al.* 2016).

BUSCO is a tool for evaluating genome and annotation completeness based on the presence of core genes in curated taxon-specific databases (Simão *et al.* 2015). For the purposes of assessing the completeness of the updated 0-1 reference genome, BUSCO was run utilizing the Ascomycota core gene set, which is comprised of a total of 1315 core genes. Results from running BUSCO on the updated 0-1 genome resulted in 97.8% of genes being present from the core Ascomycota gene set. Of the genes present relating to the core Ascomycota gene set, 97.7% were complete, 0.8% were fragmented, and 1.6% were missing. These results compare well with four other previously sequenced Dothideomycetes including *P. tritici-repentis Pt-1C-BFP* (97.6%), *P. nodorum SN15* (97.4%), and *L. maculans JN3* (97.6%) (Table 4).

**Table 4:** BUSCO analysis on assembly and annotations

Species	Isolate	BUSCO Library*^a^*	Complete*^b^*	Fragmented*^c^*	Missing*^d^*
*P. teres*	*0-1*	Ascomycota	1285	10	20
*P. tritici repentis*	*Pt-1C-BFP*	Ascomycota	1283	15	17
*P. nodorum*	*SN15*	Ascomycota	1280	17	18
*L. maculans*	*JN3*	Ascomycota	1284	10	21

a Busco contains custom curated libraries for different taxa. The Ascomycota library consists of 1315 genes and was used in this analysis.

b Complete Busco Ascomycota genes identified.

c Partially identified Busco Ascomycota genes.

d Missing Busco Ascomycota genes.

### Functional analysis and evidence of a two-speed genome

The host–pathogen interaction is directly modulated by a suite of pathogen-secreted proteins known as effectors (Franceschetti *et al.* 2017). Effectors work to modulate plant cell physiology to facilitate pathogen infection. SignalP v4 (Petersen *et al.* 2011) predicted 1002 secretion signals from the 11,541 *P. teres* f. *teres* isolate 0-1 annotated genes (Table 3), representing the secretome of 0-1. Mature amino acid sequences (lacking secretion signals) were input into EffectorP v1 (Sperschneider *et al.* 2016) to further differentiate the effectorome from within the predicted secretome, resulting in a total of 167 proteins predicted to be effectors (Table 3). These predicted effectors are likely to be important in the barley–*P. teres* f. *teres* interaction.

**Figure 1:**
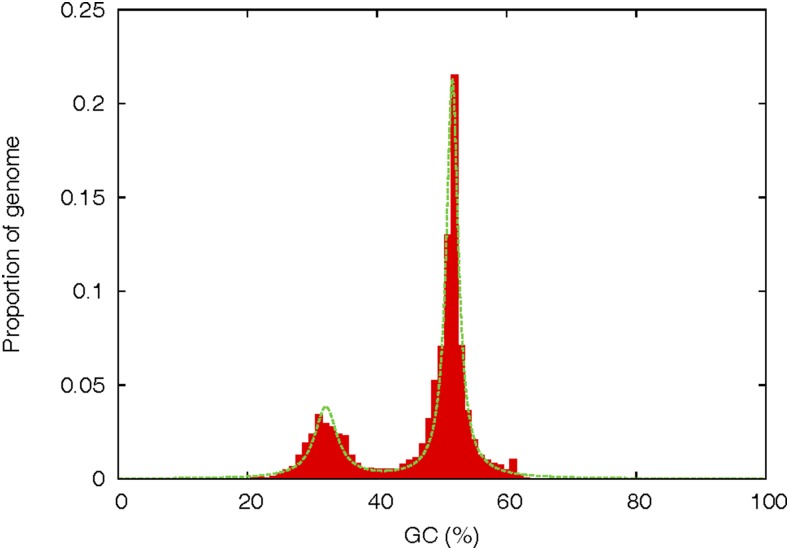
OcculterCut v1 GC% plot of the *P. teres* f. *teres* 0-1 genome. A bimodal distribution is observed in which genome segments fall into the high GC% category (41–100%) or the low GC% category (0–41%). The OcculterCut calculated cut off for distinguishing low and high GC% content in 0-1 is 41%.

Fungal plant pathogens have been shown to have bipartite compartmentalized genomes comprised of gene-rich, repeat-sparse, regions and gene-sparse, repeat-rich regions. This genomic architecture represents the “two-speed genome” model, which has been observed in a number of plant pathogens (Dong *et al.* 2015; Faino *et al.* 2016; Laurent *et al.* 2017; Rouxel and Balesdent 2017). Genes in close proximity to repeat-rich and gene-sparse regions have been shown to undergo higher rates of positive selection, indicating these compartments are evolutionarily active (Raffaele *et al.* 2010). A fungal genome defense mechanism against duplication events known as RIP may be a contributing factor in the development of AT-rich genome regions. Evolution rates of genes in close proximity to AT-rich regions could be increased through the aid of RIP (Lo Presti *et al.* 2015; Testa *et al.* 2016). The program OcculterCut v1 (Testa *et al.* 2016) was used to examine the overall GC content of the genome. The output of this analysis resulted in two distinct genome categories: one with high GC content (41–100%) and the other with low GC content (0–41%) (Figure 1 and Table 5). The high GC content portion constituted ∼75% of the genomic content, with an average gene density of 306 genes/Mb, in contrast to the low GC content region, which constitutes 24.9% of the genome with an average gene density of 72.6 genes/Mb (Table 5). This clear genomic segmentation is a classic representation of the two-speed fungal genome often seen in plant pathogens (Dong *et al.* 2015; Testa *et al.* 2016; Thomma *et al.* 2016).

**Table 5:** OcculterCut analysis of *P. teres* f. *teres*

Feature	High GC Content (>41–100%)	Low GC Content (0–41%)
Peak GC content*^a^*	51.6%	32.2%
Percentage of genome*^b^*	75.1%	24.9%
Average region length (kb)*^c^*	58.3	18.3
Number of genes in regions*^d^*	10,501	797
Gene density (genes/Mb)*^e^*	306	72.6

a Peak GC content in the elevated GC regions of the genome.

b Proportion of the genome containing a high or low GC content.

c Average length of identified GC-rich and GC-poor regions in the genome.

d Number of genes residing in GC-rich and GC-poor regions of the genome.

e Density of annotated genes within the GC-rich and GC-poor regions of the genome.

The OcculterCut analysis supports the repetitive analysis output through the RepeatModeler/RepeatMasker pipeline. This repetitive analysis identified 26.7% of the 0-1 genome as being interspersed repeat elements, and an additional 5.0% of simple repeats. This equates to roughly 32% of the genome being comprised of repetitive elements—a greater number compared to the closely related species *P. tritici-repentis* (16.7%) and *P. nodorum* (4.52%) (Manning *et al.* 2013; Syme *et al.* 2016). The most numerous repetitive element classes annotated were the LTR-Gypsy elements. comprising 9.28% of the genome. and DNA-TcMar-Fot1 elements. at 5.38% of the genome. with an additional 7.81% of the genome belonging to unclassified transposable elements (Table S1 in File S1).

RIP is a genomic defense mechanism against transposons that has been identified in a number of fungal species (Singer and Selker 1995; Dean *et al.* 2005; Idnurm and Howlett 2003; Manning *et al.* 2013; Syme *et al.* 2016). RIP involves C:G nucleotide transitions to T:A nucleotides, and affects sequences with ∼80% identity over at least 400 bp in length, creating a bias toward TpA dinucleotides over CpA dinucleotides in RIP-affected areas (Hane and Oliver 2008). RIPCAL RIP indices were calculated for the top five annotated repeat families (Table S2 in File S1), and compared to a set of randomly extracted DNA sequences of the same size range of the 0-1 genome. RIP indices that show evidence of RIP were defined as values of TpA/ApT > 2.0 and/or (CpA+TpG)/(ApC+GpT) < 0.7 (Galagan *et al.* 2003). Using these criteria, all five repeat families show evidence of RIP with (CpA+TpG)/(ApC+GpT) < 0.7, but none of the five repeat families show evidence of RIP with TpA/ApT > 2.0 (Table S2 in File S1). RIPCAL alignment “degenerative consensus” analysis of the five repeat families indicated a RIP dominance of CpT to TpA transitions (TpG to TpA in the reverse complement) and CpT to TpT transitions (TpG to TpT in the reverse complement) (Figure S2 in File S1). Given the indication of RIP-affected sequences from only one of the common indices, and the high degree of homology observed between members of the five repeat families examined, it would seem that RIP is not an efficient process in the *P. teres* f. *teres* isolate 0-1 genome. This is further supported by the increased repetitive content of the 0-1 genome relative to closely related species, and reflects similar results observed in *P. tritici-repentis* (Manning *et al.* 2013), which concluded that, if RIP is functional, the efficiency is low.

### Whole-genome alignment

Using a combination of MUMmer v3.0 (Delcher *et al.* 2003) and Bedtools v2.26.0 (Quinlan and Hall 2010), whole genome alignments were calculated and compared between the first draft assembly of *P. teres* f. *teres* isolate 0-1 and the newly assembled reference genome of 0-1. Alignments between the first draft assembly and the 12 reference scaffolds resulted in coverages ranging from 60.5 to 80.1% for the 12 reference scaffolds, equating to an average of 27.2% missing sequence between the first draft assembly and the current 12 reference scaffolds. This amount of missing data correlates well with the amount of repetitive elements detected in the genome (32%), and, in fact, 67.1% of the 27.2% missing sequence of the first draft assembly contains an annotated repeat element in the new reference genome of isolate 0-1 (Figure S2 in File S1).

The correlation between missing sequence and repetitive elements adds support to previous observations that short-read technologies struggle to span low complexity regions (Ekblom and Wolf 2014), and highlights the usefulness of long-read technologies such as PacBio (Goodwin *et al.* 2016). With regards to host–pathogen interactions, long-read technologies will aid in understanding effector genes, which can be difficult to identify as they are known to associate with low complexity repeat regions (Thomma *et al.* 2016). Long-read technologies present the best method for sequencing and assembling the genomes of fungal plant pathogen species with the goal of understanding the host–pathogen interactions.

### Conclusion

Here, we present an updated assembly and annotation of the barley pathogenic fungus *P. teres* f. *teres* reference isolate 0-1. This improved assembly and annotation provides a higher resolution assembly of the *P. teres* f. *teres* 0-1 reference genome and annotation, which now includes a large proportion of the repetitive content within the genome that have been shown to be of evolutionary importance to plant pathogens (Dong *et al.* 2015). This data set will be particularly useful in investigating effector genes that have been reported to reside in, and proximal to, evolutionarily active repetitive regions of the genome.

## Supplementary Material

Supplemental material is available online at www.g3journal.org/lookup/suppl/doi:10.1534/g3.117.300196/-/DC1.
