# Supplementary material for: Reference Assembly and Annotation of the Pyrenophora teres f. teres Isolate 0-1
Source: G3 (Bethesda). 2017 Nov 21;8(1):1–8. doi: 10.1534/g3.117.300196 (PMC5765338; doi:10.1534/g3.117.300196)
Supplement: Supplementary file 1 [file 1FileS1.pdf]

Reference assembly and annotation of the *Pyrenophora teres* f. *teres* isolate 0-1

N. A. Wyatt[*,†], J. K. Richards[†], R. S. Brueggeman[*,†], T. L. Friesen[*,†,‡]

[†]Department of Plant Pathology, North Dakota State University, Fargo, ND USA

[*]Genomics and Bioinformatics Program, North Dakota State University, Fargo, ND USA

[‡]Cereal Crops Research Unit, Red River Valley Agricultural Research Center, USDA-ARS, Fargo, ND USA

Supplementary files

Figure S1 pages 2-13

Figure S2 pages 14-19

Figure S3 pages 20-21

Table S1 page 22

Table S2 page 23

Figure S1 ALLMAPS reconstructed scaffolds. Each scaffold is represented with a side-by-side panel. The left panel contains alignments between scaffolds and linkage groups and contig boundaries within scaffolds are denoted by alternating white and grey shading. The right side panel is a scatter plot of the physical location of markers within the scaffold (x-axis) and the genetic position within the linkage group (y-axis). Contig boundaries are denoted by vertical yellow lines within the right panel scatter plots. The ρ-value at the top of each scatter plot represents the Pearson correlation coefficient with values ranging from -1 to 1 (values of -1 and 1 represent perfect collinearity).

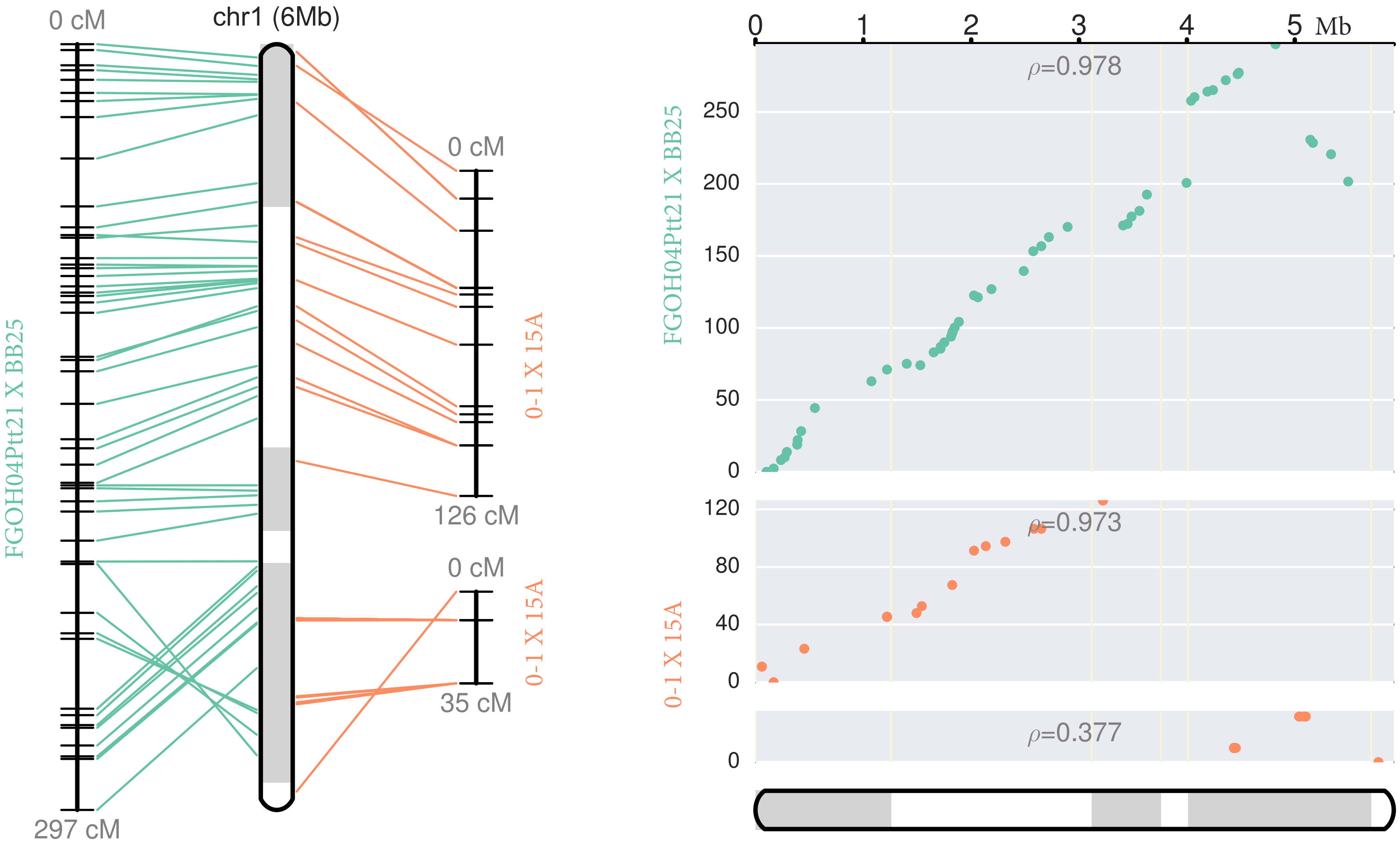

0 cM
chr1 (6Mb)
297 cM
FGOH04Ptt21 X BB25
0 cM
126 cM
0-1 X 15A
0 cM
35 cM
0-1 X 15A
0
1
2
3
4
5
Mb
ρ=0.978
250
200
150
100
50
0
FGOH04Ptt21 X BB25
120
80
40
0
ρ=0.973
0-1 X 15A
ρ=0.377
0

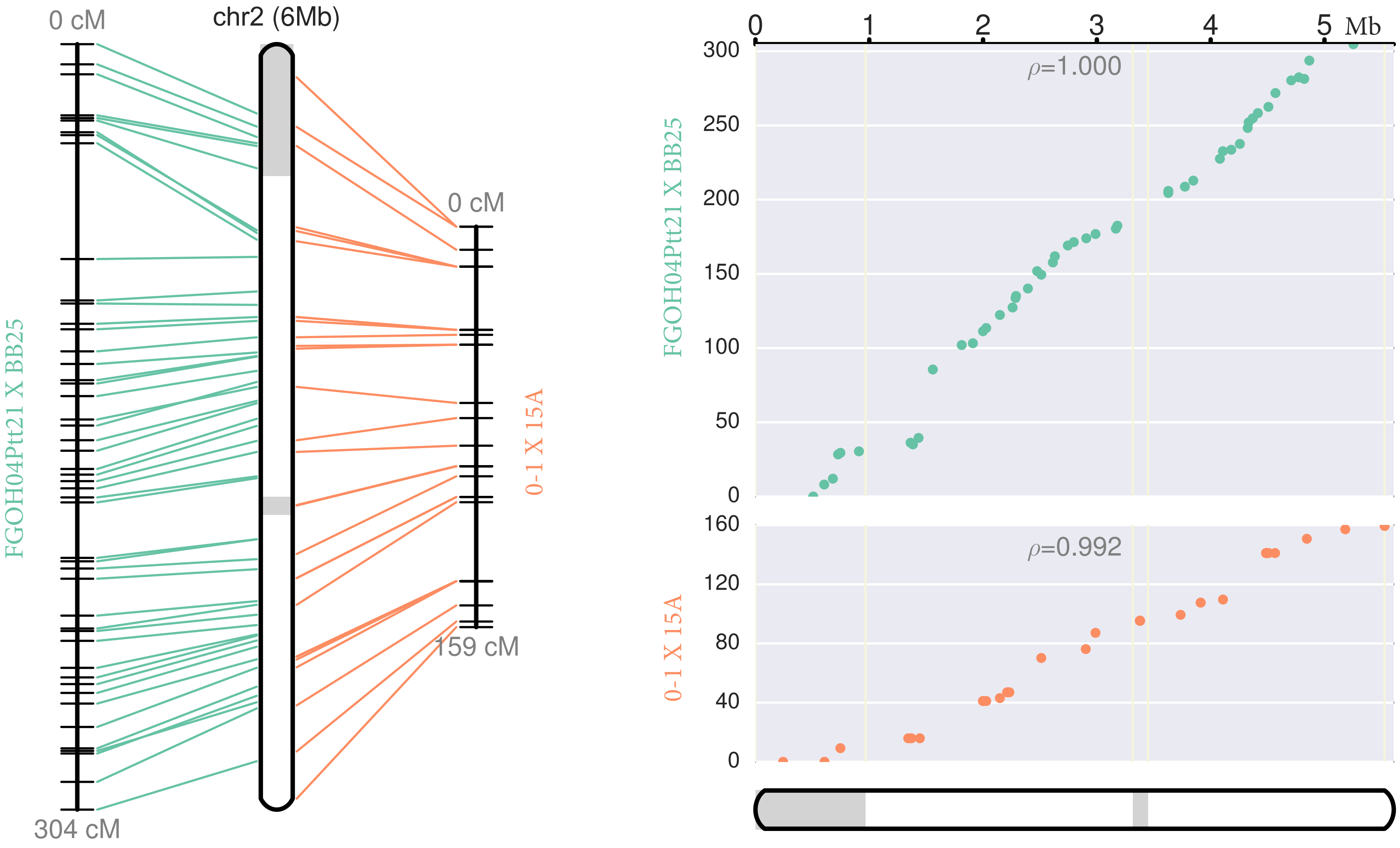

0 cM
chr2 (6Mb)
304 cM
FGOH04Ptt21 X BB25
0 cM
159 cM
0-1 X 15A
0
1
2
3
4
5
Mb
ρ=1.000
ρ=0.992
300
250
200
150
100
50
0
160
120
80
40
0
FGOH04Ptt21 X BB25
0-1 X 15A

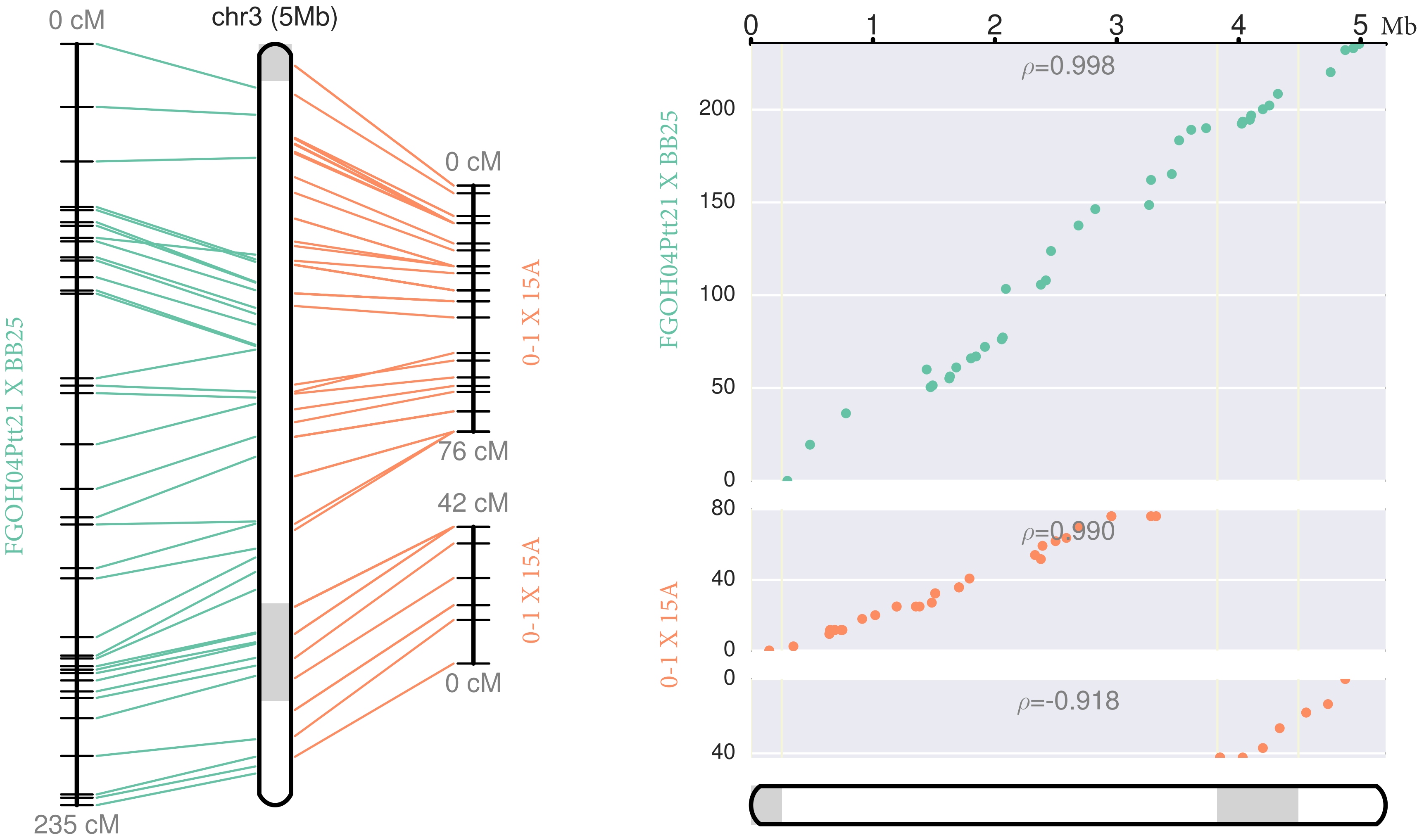

0 cM
chr3 (5Mb)
235 cM
FGOH04Ptt21 X BB25
0 cM
76 cM
42 cM
0 cM
0-1 X 15A
0-1 X 15A
0 1 2 3 4 5 Mb
ρ=0.998
ρ=0.990
ρ=-0.918
200
150
100
50
0
80
40
0
0
40
FGOH04Ptt21 X BB25
0-1 X 15A

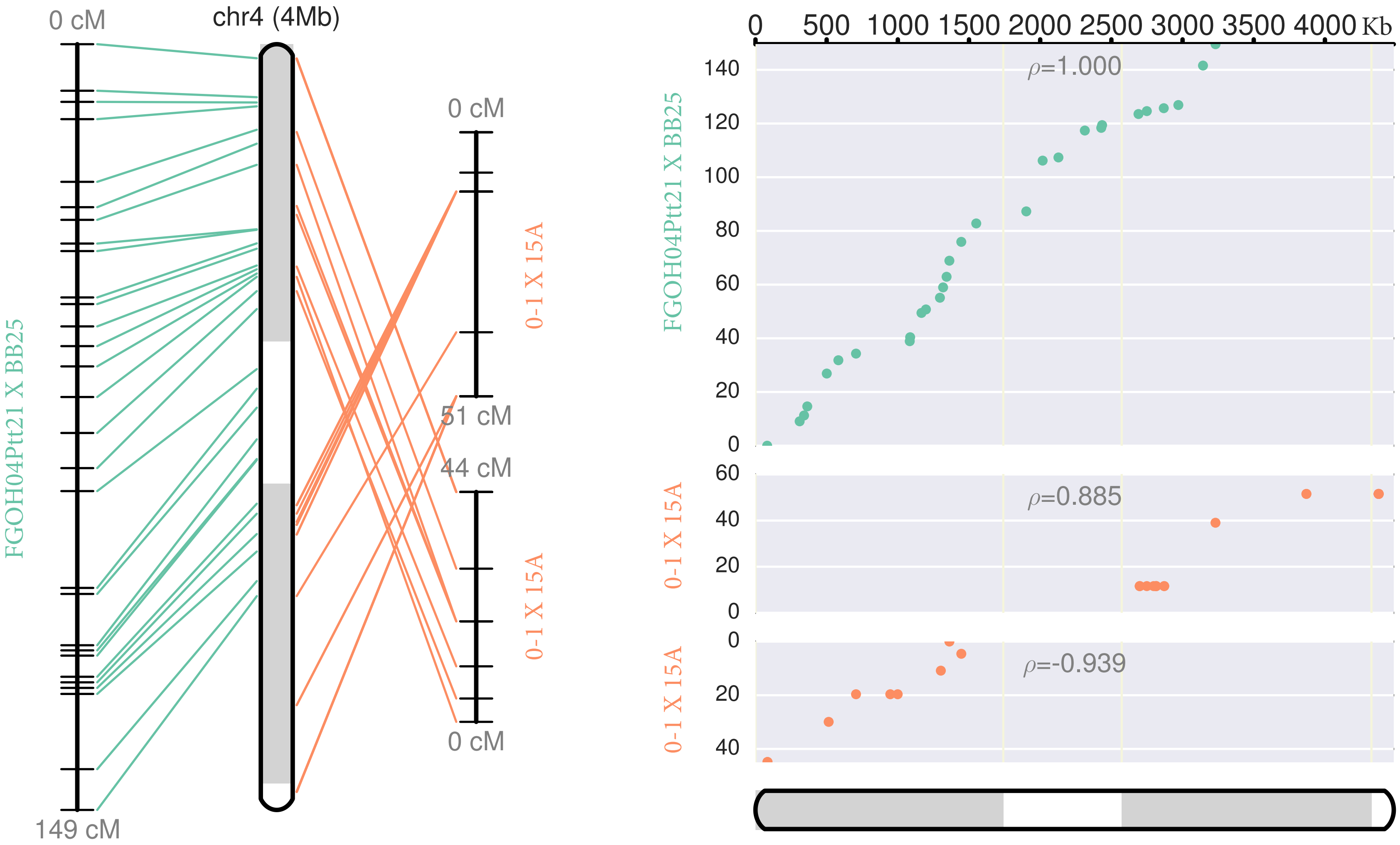
0 cM
chr4 (4Mb)
0 cM
51 cM
44 cM
0 cM
149 cM
FGOH04Ptt21 X BB25
0-1 X 15A
0-1 X 15A
0 500 1000 1500 2000 2500 3000 3500 4000 Kb
ρ=1.000
ρ=0.885
ρ=-0.939
FGOH04Ptt21 X BB25
0-1 X 15A
0-1 X 15A
140
120
100
80
60
40
20
0
60
40
20
0
0
20
40

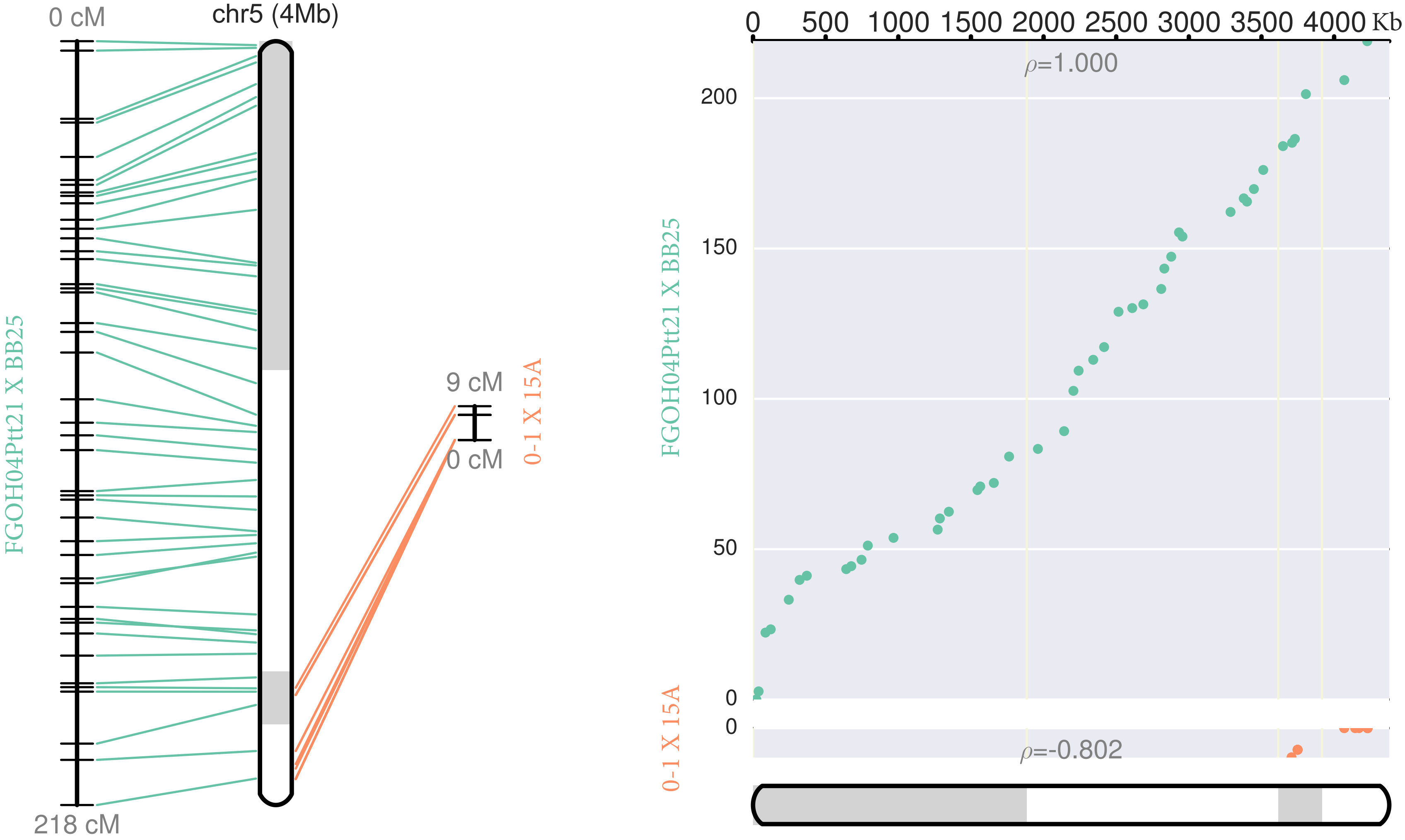

0 cM
chr5 (4Mb)
218 cM
FGOH04Ptt21 X BB25
9 cM
0 cM
0-1 X 15A
0
500
1000
1500
2000
2500
3000
3500
4000
Kb
ρ=1.000
200
150
100
50
0
0
ρ=-0.802
FGOH04Ptt21 X BB25
0-1 X 15A

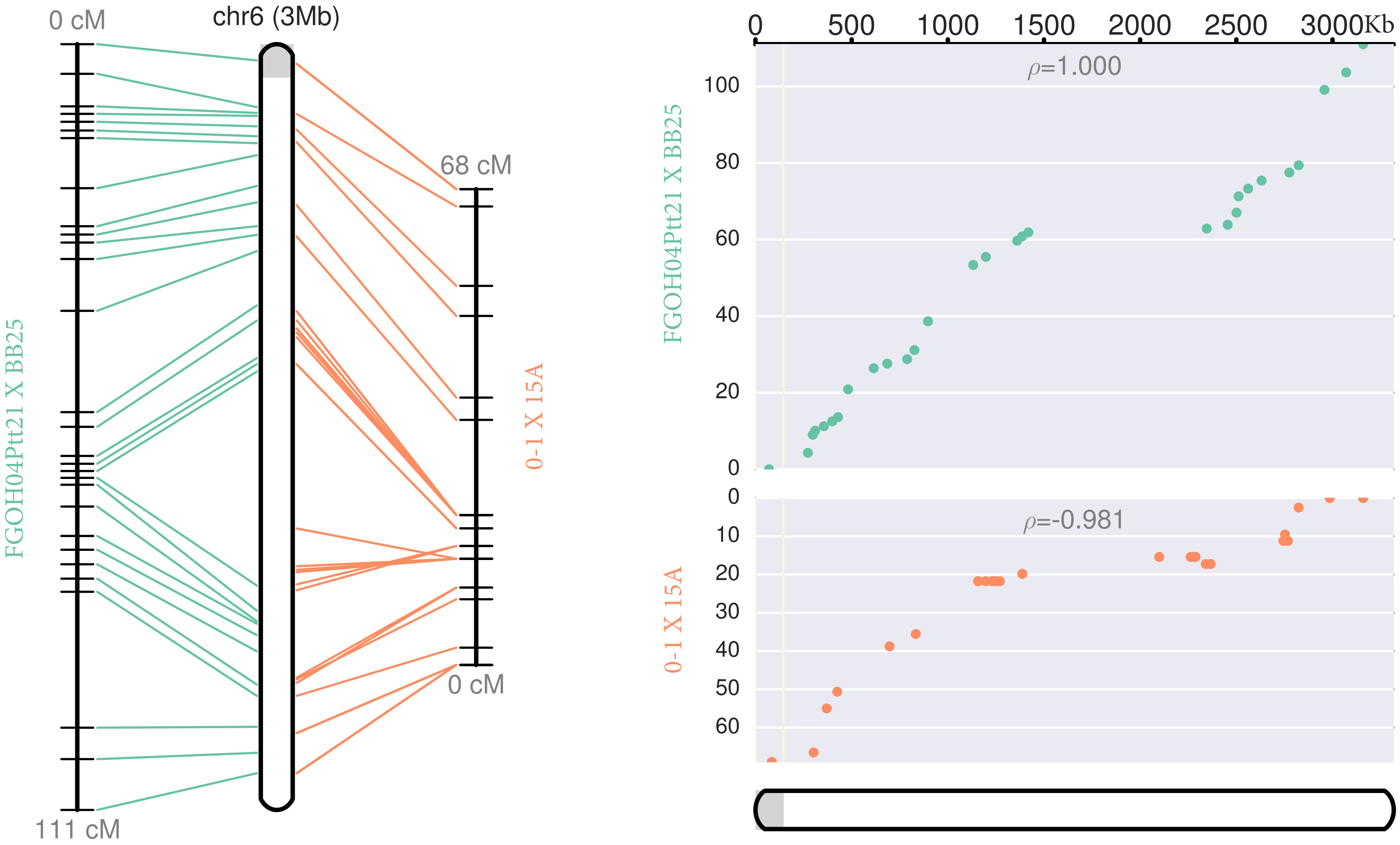
0 cM
chr6 (3Mb)
68 cM
0 cM
111 cM
FGOH04Ptt21 X BB25
0-1 X 15A
0
500
1000
1500
2000
2500
3000Kb
ρ=1.000
ρ=-0.981
100
80
60
40
20
0
0
10
20
30
40
50
60

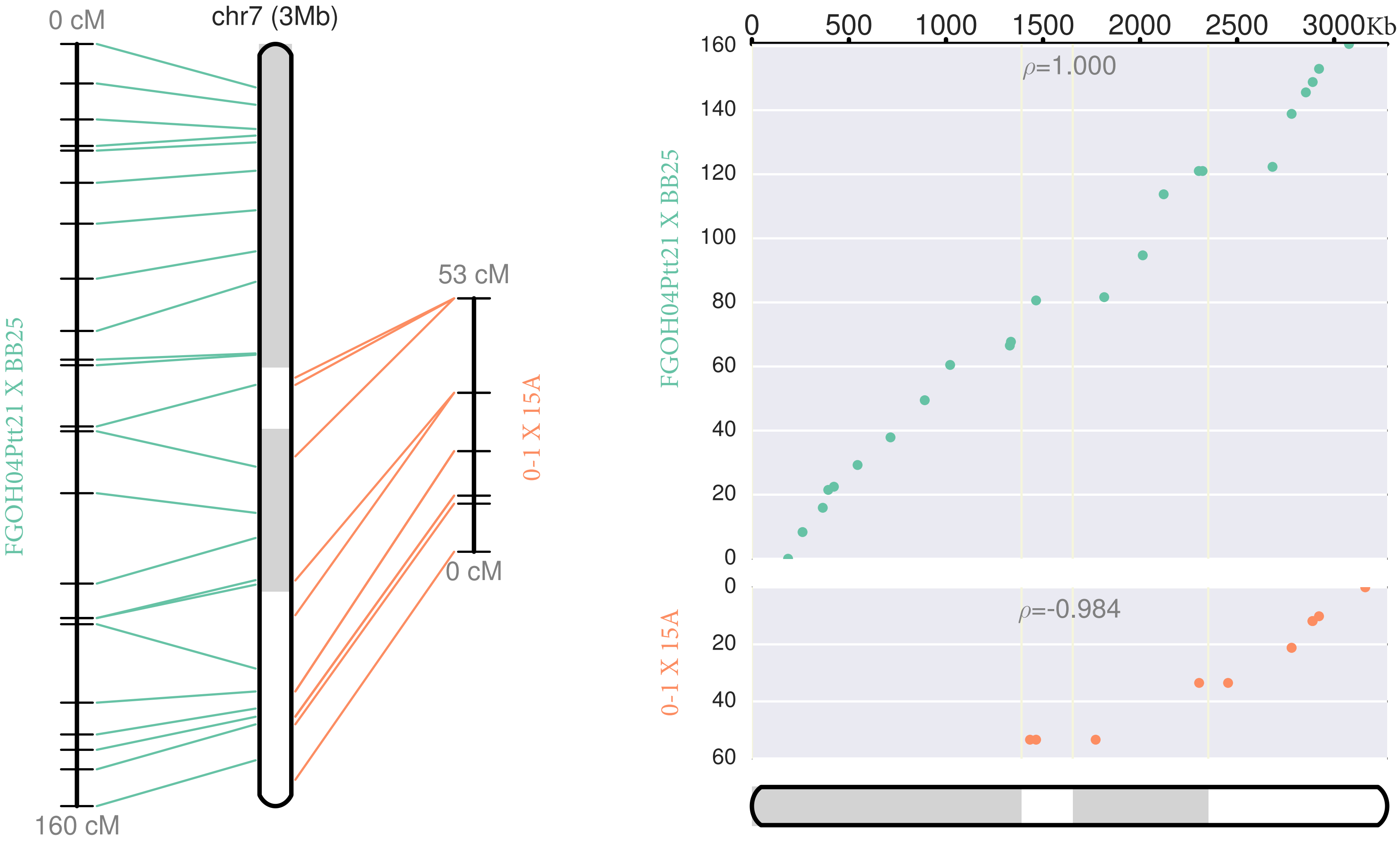

0 cM
chr7 (3Mb)
160 cM
FGOH04Ptt21 X BB25
53 cM
0 cM
0-1 X 15A
0
500
1000
1500
2000
2500
3000Kb
ρ=1.000
ρ=-0.984
160
140
120
100
80
60
40
20
0
FGOH04Ptt21 X BB25
0
20
40
60
0-1 X 15A

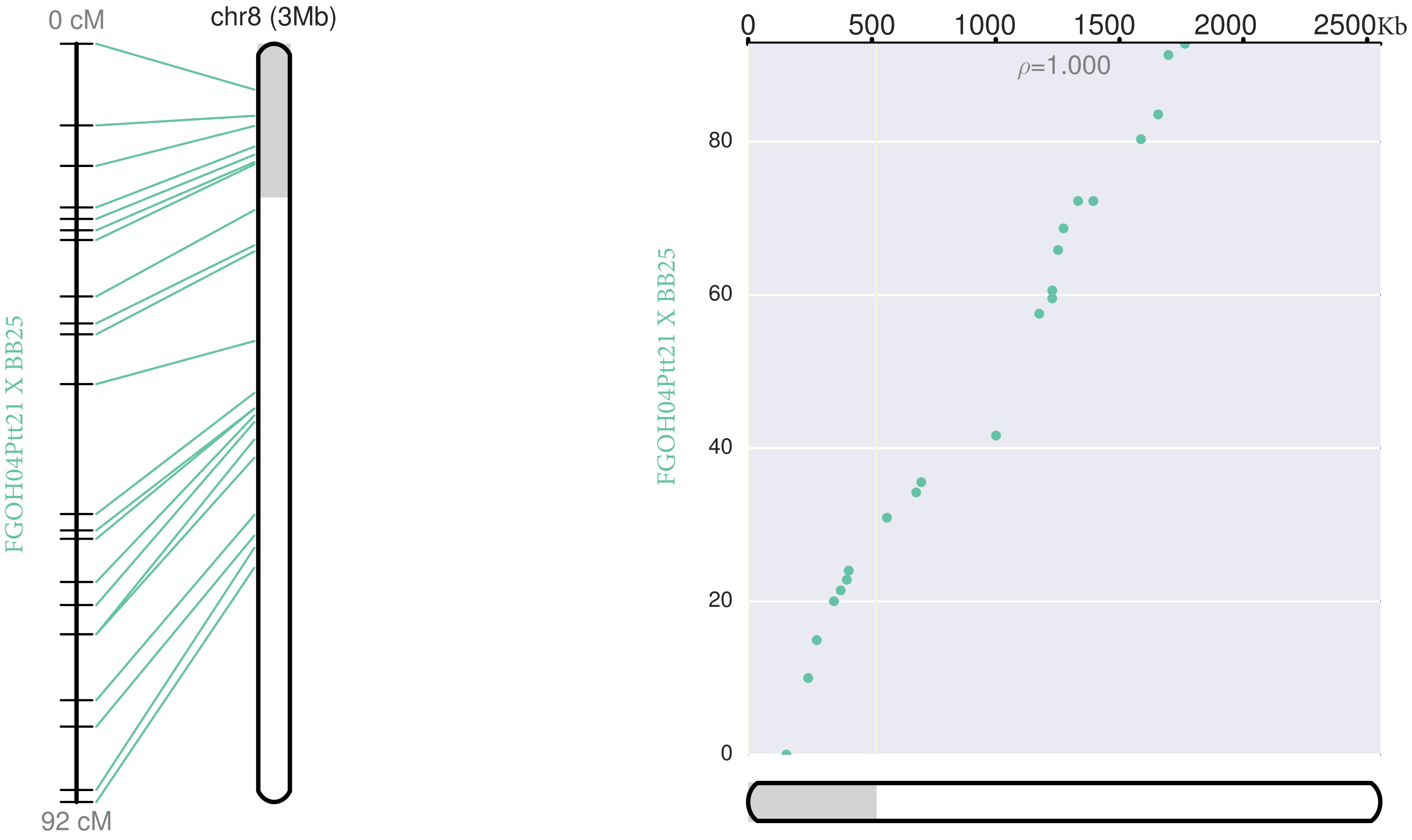

0 cM
chr8 (3Mb)
92 cM
FGOH04Ptt21 X BB25
0
500
1000
1500
2000
2500Kb
ρ=1.000
80
60
40
20
0
FGOH04Ptt21 X BB25

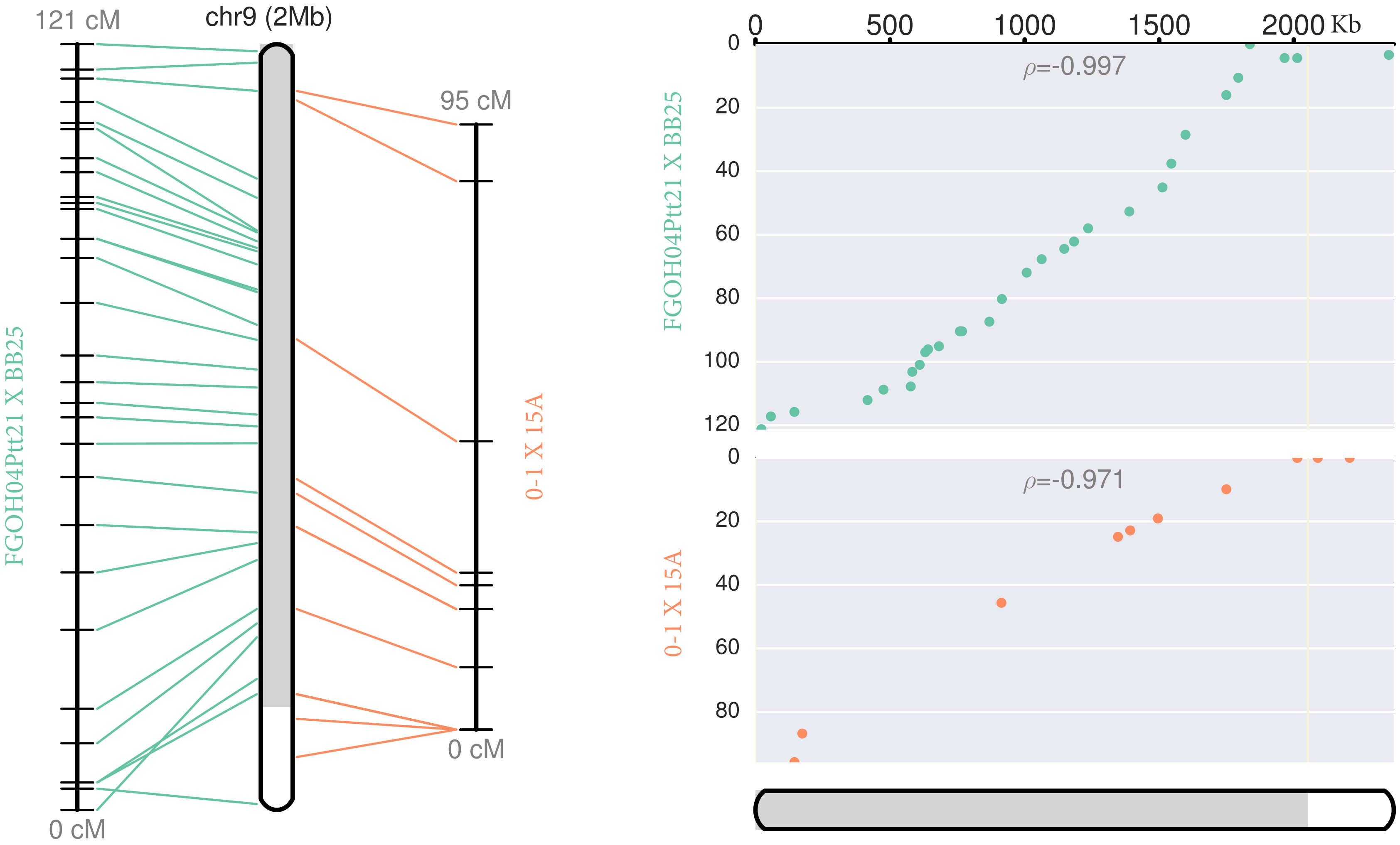

121 cM
chr9 (2Mb)
95 cM
FGOH04Ptt21 X BB25
0-1 X 15A
0 cM
0 cM
0
500
1000
1500
2000
Kb
ρ=-0.997
ρ=-0.971
0
20
40
60
80
100
120
0
20
40
60
80

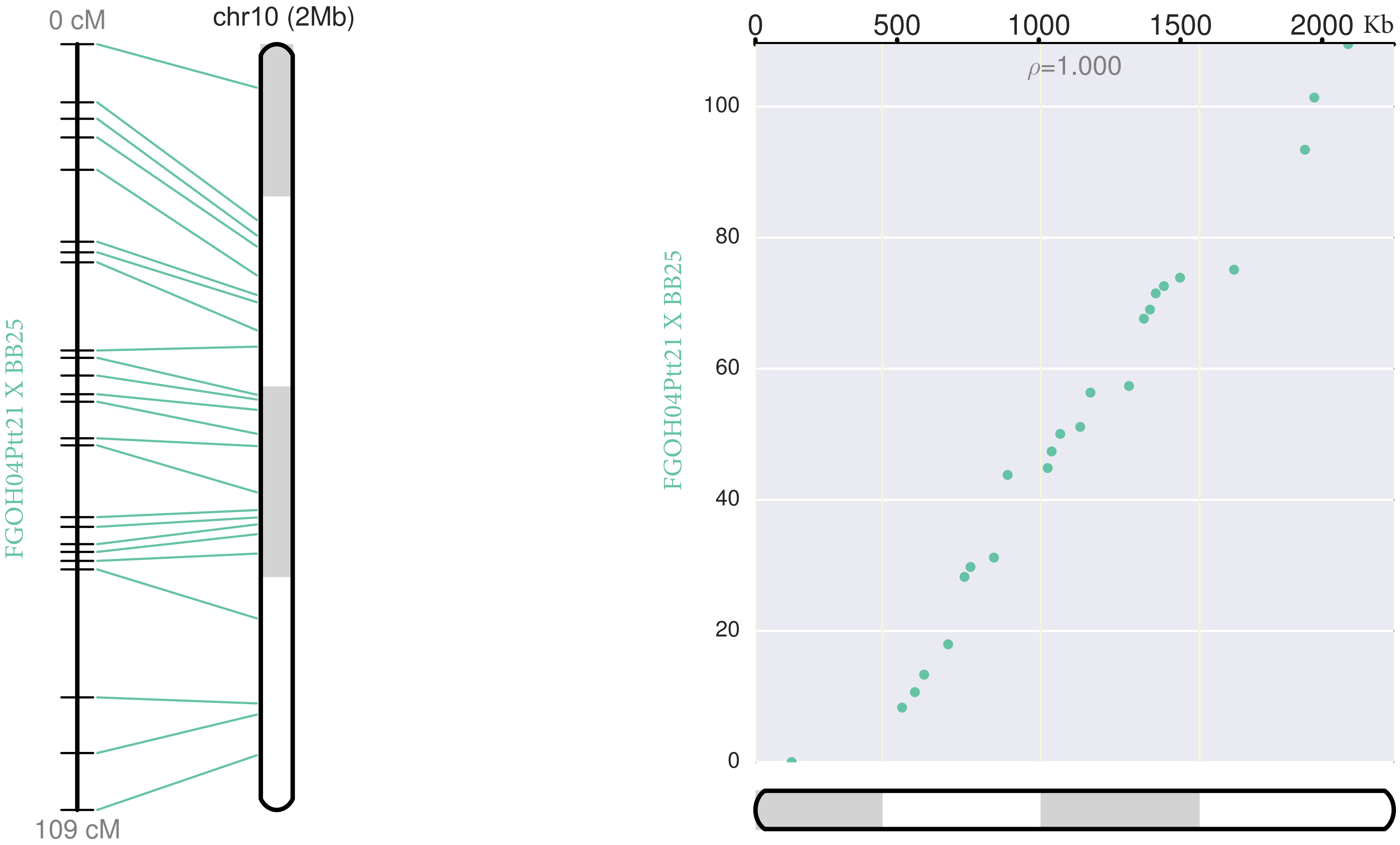
0 cM
chr10 (2Mb)
FGOH04Ptt21 X BB25
109 cM
0
500
1000
1500
2000
Kb
ρ=1.000
100
80
60
40
20
0
FGOH04Ptt21 X BB25

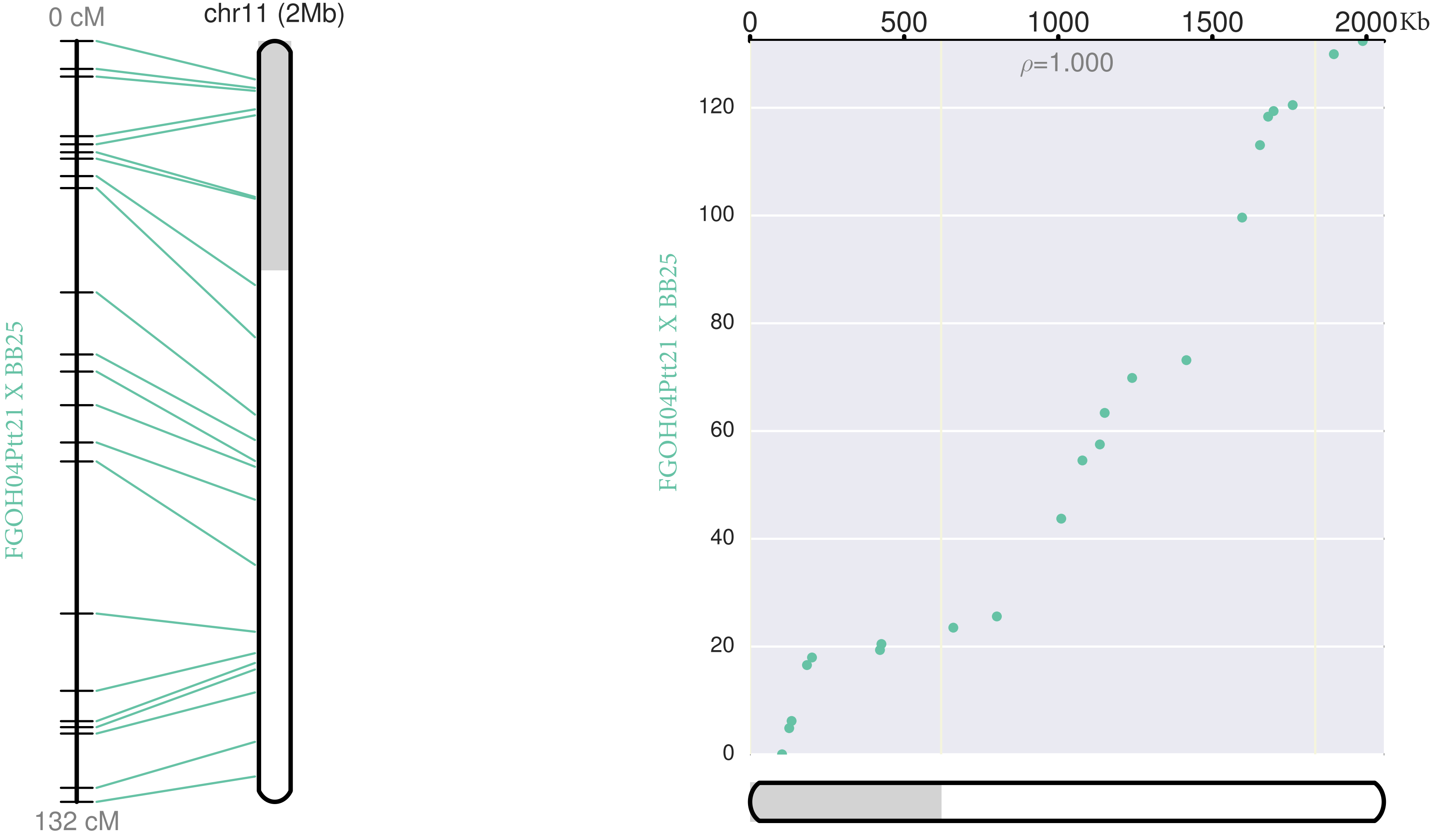
0 cM
chr11 (2Mb)
FGOH04Ptt21 X BB25
132 cM
0
500
1000
1500
2000
Kb
ρ=1.000
120
100
80
60
40
20
0
FGOH04Ptt21 X BB25

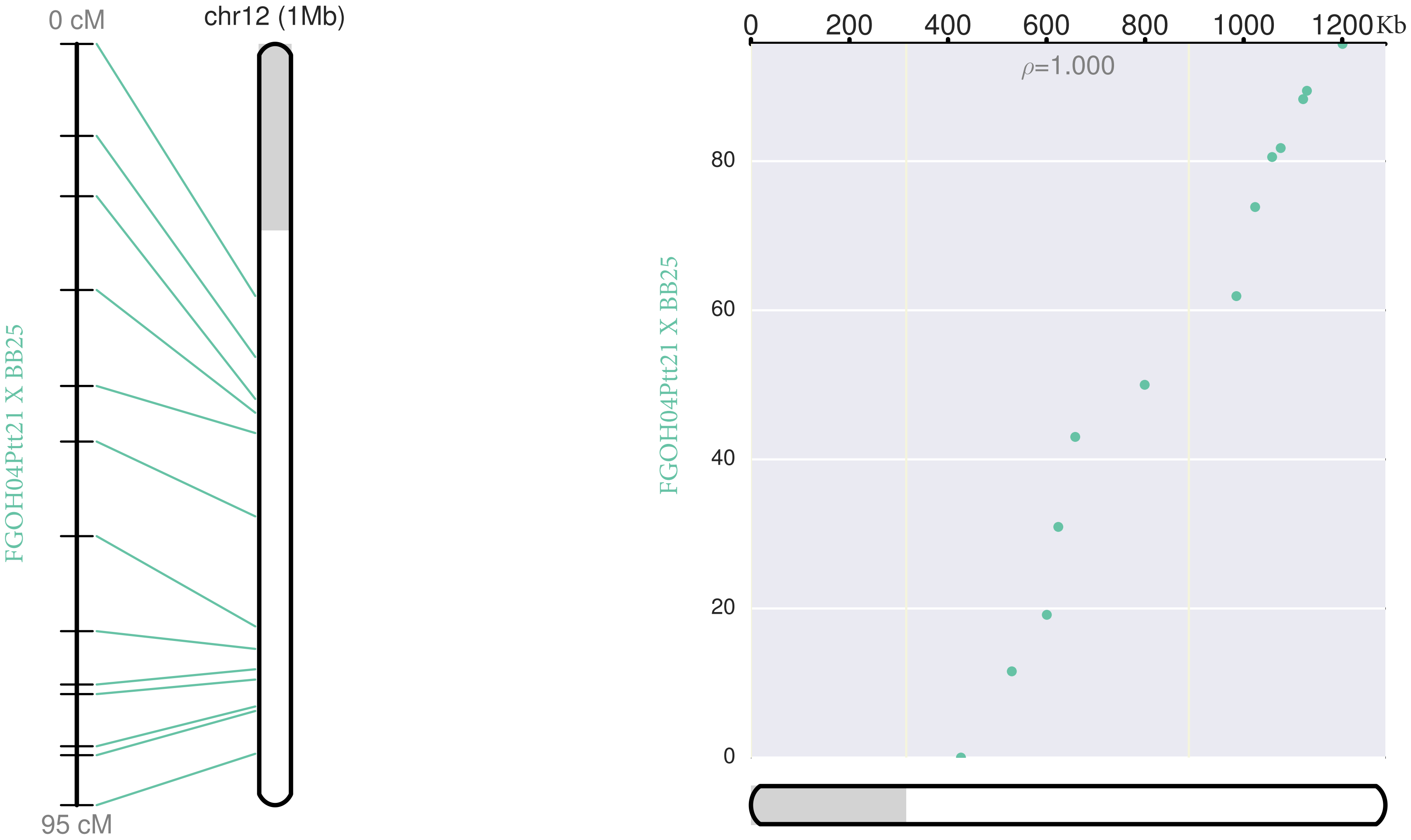

0 cM
chr12 (1Mb)
FGOH04Ptt21 X BB25
95 cM
0
200
400
600
800
1000
1200
Kb
ρ=1.000
80
60
40
20
0
FGOH04Ptt21 X BB25

Figure S2 RIPCAL alignments of the five most numerous repeat families in P. teres f. teres isolate 0-1 (MOLLY_SN, GYPSY2_CH, GYPSY3_CH, GYPSY4_CH, and LMR1_LM). The colored bars at the top of the figure represent an alignment of the copies of the repeat, titled above each plot. Red bars represent likely CpA to TpA transitions, blue bars represent likely CpC to TpC transitions, green bars represent likely CpG to TpG transitions, and turquoise bars represent CpT to TpT transistions; gray bars, black bars and white bars represent mismatches, matches, and gaps relative to the consensus sequence, respectively. Graphs below show total number of mutations for each potential type of RIP (i.e., CpA to TpA, CpC to TpC, CpG to TpG, CpT to TpT) at each site in the alignment.

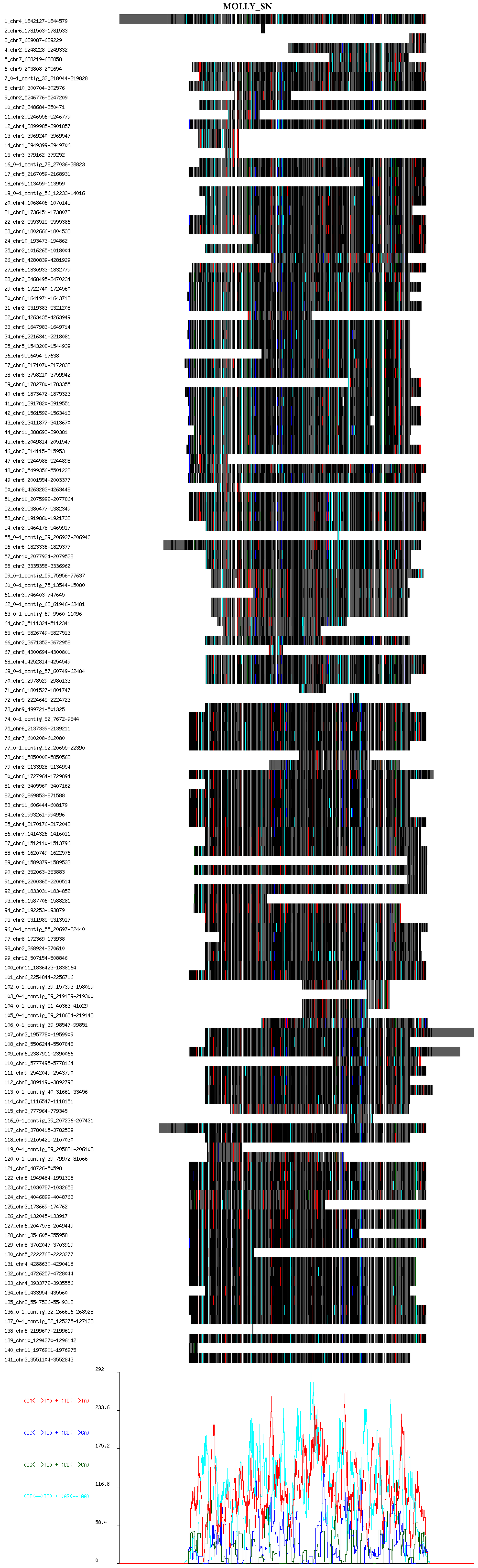
MOLLY_SN
1_chr4_1842127-1844579
2_chr6_1781503-1781533
3_chr7_689087-689229
4_chr2_5248228-5249332
5_chr7_688219-688858
6_chr5_203808-205654
7_0-1_contig_32_218044-219828
8_chr10_300704-302576
9_chr2_5246776-5247209
10_chr2_348684-350471
11_chr2_5246556-5246779
12_chr4_3899985-3901857
13_chr1_3969240-3969547
14_chr1_3949399-3949706
15_chr3_379162-379252
16_0-1_contig_78_27036-28823
17_chr5_2167059-2168931
18_chr9_113459-113959
19_0-1_contig_56_12233-14016
20_chr4_1068406-1070145
21_chr8_1736451-1738072
22_chr2_5553515-5555386
23_chr6_1802666-1804538
24_chr10_193473-194862
25_chr2_1016265-1018004
26_chr8_4280839-4281929
27_chr6_1830933-1832779
28_chr2_3468495-3470234
29_chr6_1722740-1724560
30_chr6_1641971-1643713
31_chr2_5319383-5321208
32_chr8_4263435-4263949
33_chr6_1647983-1649714
34_chr6_2216341-2218081
35_chr5_1543208-1544939
36_chr9_56454-57638
37_chr6_2171070-2172832
38_chr8_3758210-3759942
39_chr6_1782780-1783355
40_chr6_1873472-1875323
41_chr1_3917820-3919551
42_chr6_1561592-1563413
43_chr2_3411877-3413670
44_chr11_388693-390381
45_chr6_2049814-2051547
46_chr2_314115-315953
47_chr2_5244588-5244898
48_chr2_5499356-5501228
49_chr6_2001554-2003377
50_chr8_4263283-4263448
51_chr10_2075992-2077864
52_chr2_5380477-5382349
53_chr6_1919860-1921732
54_chr2_5464178-5465917
55_0-1_contig_39_206927-206943
56_chr6_1823336-1825377
57_chr10_2077924-2079528
58_chr2_3335358-3336962
59_0-1_contig_59_75956-77637
60_0-1_contig_75_13544-15080
61_chr3_746403-747645
62_0-1_contig_63_61946-63481
63_0-1_contig_69_9560-11096
64_chr2_5111324-5112341
65_chr1_5826749-5827513
66_chr2_3671352-3672958
67_chr8_4300694-4300801
68_chr4_4252814-4254549
69_0-1_contig_57_60749-62484
70_chr1_2978529-2980133
71_chr6_1801527-1801747
72_chr5_2224645-2224723
73_chr9_499721-501325
74_0-1_contig_52_7672-9544
75_chr6_2137339-2139211
76_chr7_600208-602080
77_0-1_contig_52_20655-22390
78_chr1_5850008-5850563
79_chr2_5133928-5134954
80_chr6_1727964-1729894
81_chr2_3405560-3407162
82_chr2_869853-871588
83_chr11_606444-608179
84_chr2_993261-994996
85_chr4_3170176-3172048
86_chr7_1414326-1416011
87_chr6_1512110-1513796
88_chr6_1620749-1622576
89_chr6_1589379-1589533
90_chr2_352063-353883
91_chr6_2200365-2200514
92_chr6_1833031-1834852
93_chr6_1587706-1588281
94_chr2_192253-193879
95_chr2_5311985-5313517
96_0-1_contig_55_20697-22440
97_chr8_172369-173938
98_chr2_268924-270610
99_chr12_507154-508846
100_chr11_1836423-1838164
101_chr6_2254844-2256716
102_0-1_contig_39_157393-158059
103_0-1_contig_39_219139-219300
104_0-1_contig_51_40363-41029
105_0-1_contig_39_218634-219148
106_0-1_contig_39_98547-99851
107_chr3_1957780-1959909
108_chr2_5506244-5507848
109_chr6_2387911-2390066
110_chr1_5777495-5778164
111_chr9_2542049-2543790
112_chr8_3891190-3892792
113_0-1_contig_40_31661-33456
114_chr2_1116547-1118151
115_chr3_777964-779345
116_0-1_contig_39_207236-207431
117_chr8_3780415-3782539
118_chr9_2105425-2107030
119_0-1_contig_39_205831-206108
120_0-1_contig_39_79972-81066
121_chr8_48726-50598
122_chr6_1949484-1951356
123_chr2_1030787-1032658
124_chr1_4046899-4048763
125_chr3_173669-174762
126_chr8_132045-133917
127_chr6_2047578-2049449
128_chr1_354605-355958
129_chr8_3702047-3703919
130_chr5_2222768-2223277
131_chr4_4288630-4290416
132_chr1_4726257-4728044
133_chr4_3933772-3935556
134_chr5_433954-435560
135_chr2_5547526-5549312
136_0-1_contig_32_266656-268528
137_0-1_contig_32_125275-127133
138_chr6_2199607-2199619
139_chr10_1294270-1296142
140_chr11_1976901-1976975
141_chr3_3551104-3552843
292
(CA<-->TA) + (TG<-->TA)
233.6
(CC<-->TC) + (GG<-->GA)
175.2
(CG<-->TG) + (CG<-->CA)
116.8
(CT<-->TT) + (AG<-->AA)
58.4
0

GYPSY-2_CH

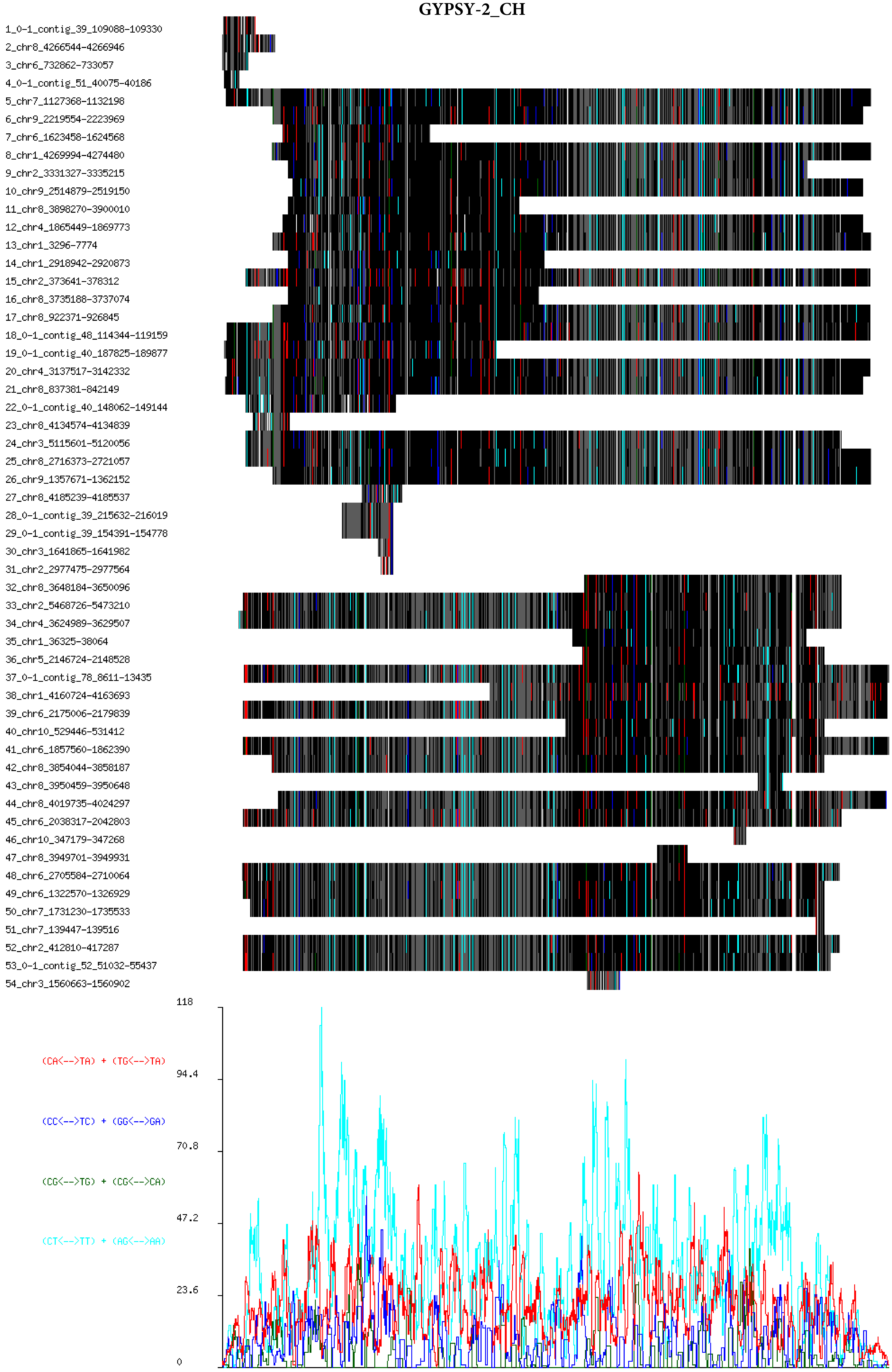
1_0-1_contig_39_109088-109330
2_chr8_4266544-4266946
3_chr6_732862-733057
4_0-1_contig_51_40075-40186
5_chr7_1127368-1132198
6_chr9_2219554-2223969
7_chr6_1623458-1624568
8_chr1_4269994-4274480
9_chr2_3331327-3335215
10_chr9_2514879-2519150
11_chr8_3898270-3900010
12_chr4_1865449-1869773
13_chr1_3296-7774
14_chr1_2918942-2920873
15_chr2_373641-378312
16_chr8_3735188-3737074
17_chr8_922371-926845
18_0-1_contig_48_114344-119159
19_0-1_contig_40_187825-189877
20_chr4_3137517-3142332
21_chr8_837381-842149
22_0-1_contig_40_148062-149144
23_chr8_4134574-4134839
24_chr3_5115601-5120056
25_chr8_2716373-2721057
26_chr9_1357671-1362152
27_chr8_4185239-4185537
28_0-1_contig_39_215632-216019
29_0-1_contig_39_154391-154778
30_chr3_1641865-1641982
31_chr2_2977475-2977564
32_chr8_3648184-3650096
33_chr2_5468726-5473210
34_chr4_3624989-3629507
35_chr1_36325-38064
36_chr5_2146724-2148528
37_0-1_contig_78_8611-13435
38_chr1_4160724-4163693
39_chr6_2175006-2179839
40_chr10_529446-531412
41_chr6_1857560-1862390
42_chr8_3854044-3858187
43_chr8_3950459-3950648
44_chr8_4019735-4024297
45_chr6_2038317-2042803
46_chr10_347179-347268
47_chr8_3949701-3949931
48_chr6_2705584-2710064
49_chr6_1322570-1326929
50_chr7_1731230-1735533
51_chr7_139447-139516
52_chr2_412810-417287
53_0-1_contig_52_51032-55437
54_chr3_1560663-1560902
118
94.4
70.8
47.2
23.6
0
(CA<-->TA) + (TG<-->TA)
(CC<-->TC) + (GG<-->GA)
(CG<-->TG) + (CG<-->CA)
(CT<-->TT) + (AG<-->AA)

GYPSY-3_CH

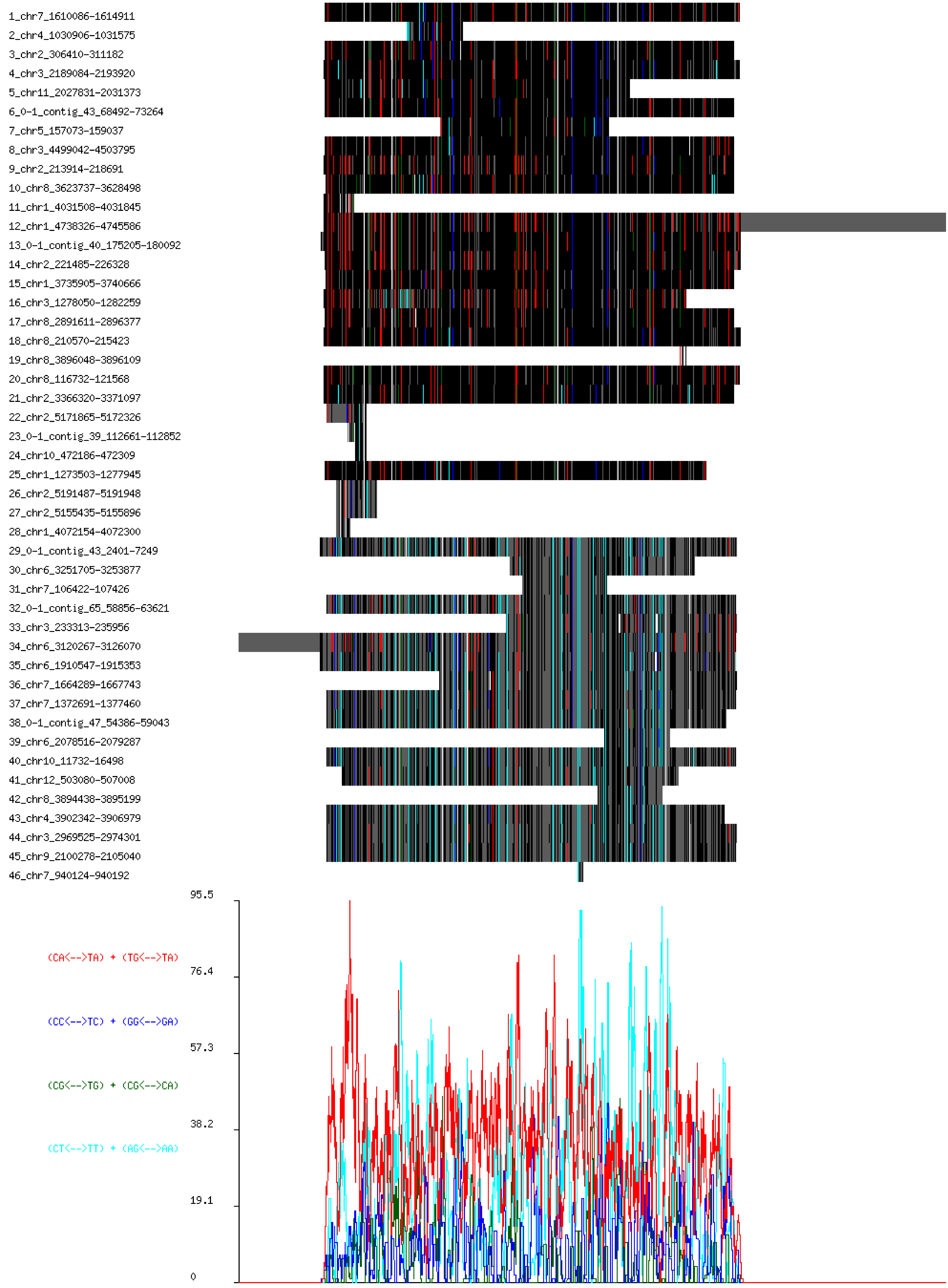
1_chr7_1610086-1614911
2_chr4_1030906-1031575
3_chr2_306410-311182
4_chr3_2189084-2193920
5_chr11_2027831-2031373
6_0-1_contig_43_68492-73264
7_chr5_157073-159037
8_chr3_4499042-4503795
9_chr2_213914-218691
10_chr8_3623737-3628498
11_chr1_4031508-4031845
12_chr1_4738326-4745586
13_0-1_contig_40_175205-180092
14_chr2_221485-226328
15_chr1_3735905-3740666
16_chr3_1278050-1282259
17_chr8_2891611-2896377
18_chr8_210570-215423
19_chr8_3896048-3896109
20_chr8_116732-121568
21_chr2_3366320-3371097
22_chr2_5171865-5172326
23_0-1_contig_39_112661-112852
24_chr10_472186-472309
25_chr1_1273503-1277945
26_chr2_5191487-5191948
27_chr2_5155435-5155896
28_chr1_4072154-4072300
29_0-1_contig_43_2401-7249
30_chr6_3251705-3253877
31_chr7_106422-107426
32_0-1_contig_65_58856-63621
33_chr3_233313-235956
34_chr6_3120267-3126070
35_chr6_1910547-1915353
36_chr7_1664289-1667743
37_chr7_1372691-1377460
38_0-1_contig_47_54386-59043
39_chr6_2078516-2079287
40_chr10_11732-16498
41_chr12_503080-507008
42_chr8_3894438-3895199
43_chr4_3902342-3906979
44_chr3_2969525-2974301
45_chr9_2100278-2105040
46_chr7_940124-940192
95.5
76.4
57.3
38.2
19.1
0
(CA<-->TA) + (TG<-->TA)
(CC<-->TC) + (GG<-->GA)
(CG<-->TG) + (CG<-->CA)
(CT<-->TT) + (AG<-->AA)

GYPSY-4_CH

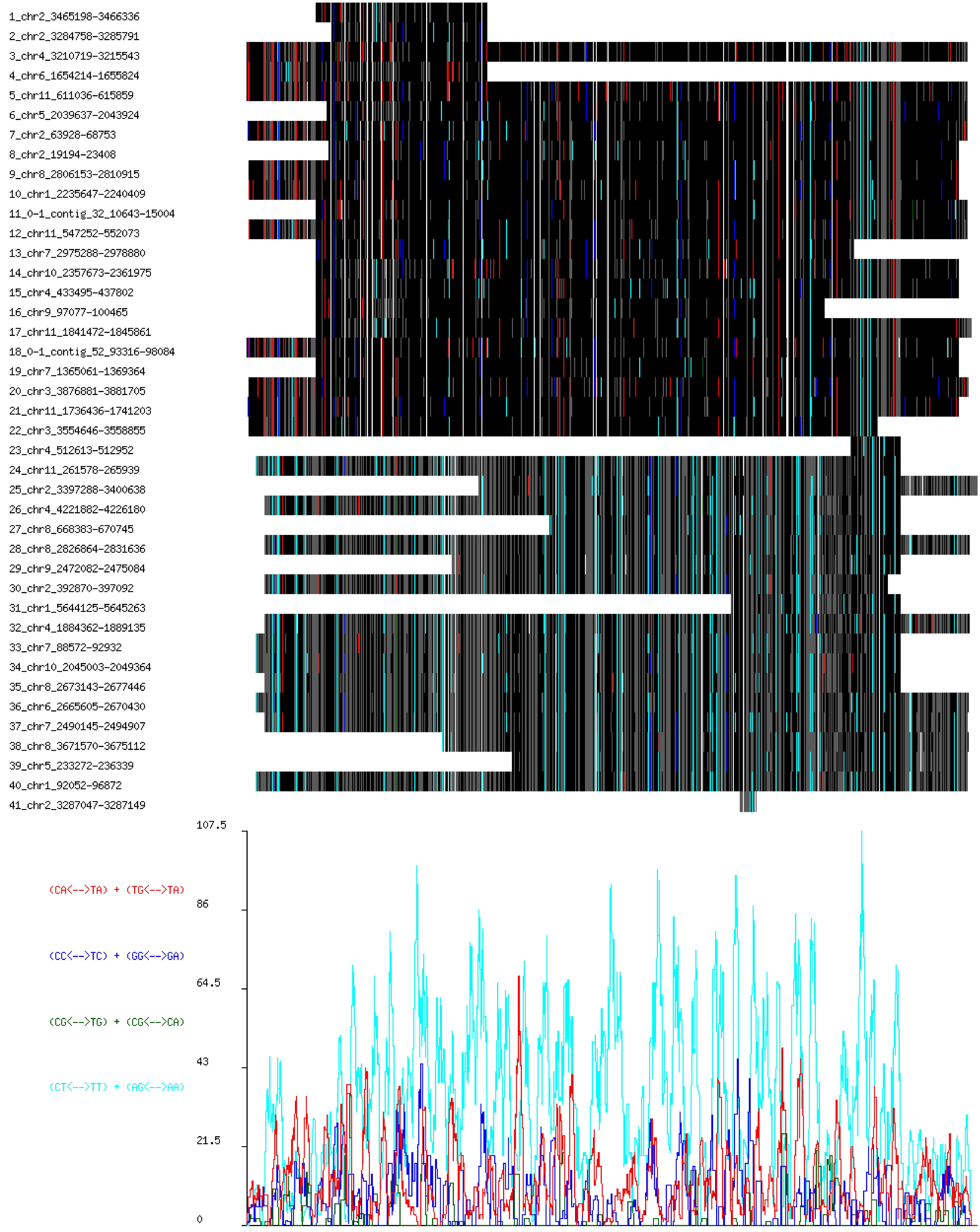

1_chr2_3465198-3466336
2_chr2_3284758-3285791
3_chr4_3210719-3215543
4_chr6_1654214-1655824
5_chr11_611036-615859
6_chr5_2039637-2043924
7_chr2_63928-68753
8_chr2_19194-23408
9_chr8_2806153-2810915
10_chr1_2235647-2240409
11_0-1_contig_32_10643-15004
12_chr11_547252-552073
13_chr7_2975288-2978880
14_chr10_2357673-2361975
15_chr4_433495-437802
16_chr9_97077-100465
17_chr11_1841472-1845861
18_0-1_contig_52_93316-98084
19_chr7_1365061-1369364
20_chr3_3876881-3881705
21_chr11_1736436-1741203
22_chr3_3554646-3558855
23_chr4_512613-512952
24_chr11_261578-265939
25_chr2_3397288-3400638
26_chr4_4221882-4226180
27_chr8_668383-670745
28_chr8_2826864-2831636
29_chr9_2472082-2475084
30_chr2_392870-397092
31_chr1_5644125-5645263
32_chr4_1884362-1889135
33_chr7_88572-92932
34_chr10_2045003-2049364
35_chr8_2673143-2677446
36_chr6_2665605-2670430
37_chr7_2490145-2494907
38_chr8_3671570-3675112
39_chr5_233272-236339
40_chr1_92052-96872
41_chr2_3287047-3287149
107.5
86
64.5
43
21.5
0
(CA<-->TA) + (TG<-->TA)
(CC<-->TC) + (GG<-->GA)
(CG<-->TG) + (CG<-->CA)
(CT<-->TT) + (AG<-->AA)

LMR1_LM

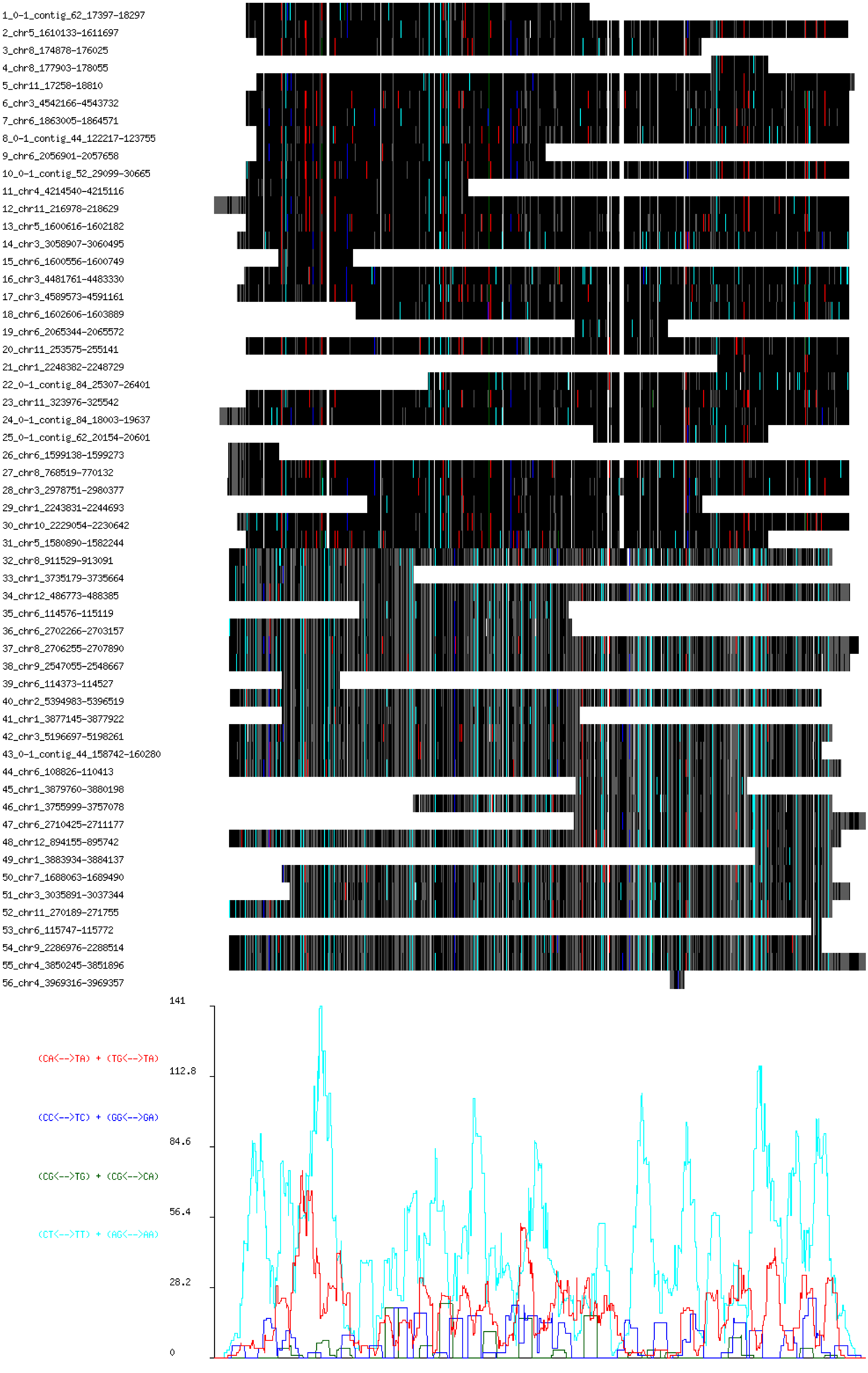

1_0-1_contig_62_17397-18297
2_chr5_1610133-1611697
3_chr8_174878-176025
4_chr8_177903-178055
5_chr11_17258-18810
6_chr3_4542166-4543732
7_chr6_1863005-1864571
8_0-1_contig_44_122217-123755
9_chr6_2056901-2057658
10_0-1_contig_52_29099-30665
11_chr4_4214540-4215116
12_chr11_216978-218629
13_chr5_1600616-1602182
14_chr3_3058907-3060495
15_chr6_1600556-1600749
16_chr3_4481761-4483330
17_chr3_4589573-4591161
18_chr6_1602606-1603889
19_chr6_2065344-2065572
20_chr11_253575-255141
21_chr1_2248382-2248729
22_0-1_contig_84_25307-26401
23_chr11_323976-325542
24_0-1_contig_84_18003-19637
25_0-1_contig_62_20154-20601
26_chr6_1599138-1599273
27_chr8_768519-770132
28_chr3_2978751-2980377
29_chr1_2243831-2244693
30_chr10_2229054-2230642
31_chr5_1580890-1582244
32_chr8_911529-913091
33_chr1_3735179-3735664
34_chr12_486773-488385
35_chr6_114576-115119
36_chr6_2702266-2703157
37_chr8_2706255-2707890
38_chr9_2547055-2548667
39_chr6_114373-114527
40_chr2_5394983-5396519
41_chr1_3877145-3877922
42_chr3_5196697-5198261
43_0-1_contig_44_158742-160280
44_chr6_108826-110413
45_chr1_3879760-3880198
46_chr1_3755999-3757078
47_chr6_2710425-2711177
48_chr12_894155-895742
49_chr1_3883934-3884137
50_chr7_1688063-1689490
51_chr3_3035891-3037344
52_chr11_270189-271755
53_chr6_115747-115772
54_chr9_2286976-2288514
55_chr4_3850245-3851896
56_chr4_3969316-3969357
141
112.8
84.6
56.4
28.2
0
(CA<-->TA) + (TG<-->TA)
(CC<-->TC) + (GG<-->GA)
(CG<-->TG) + (CG<-->CA)
(CT<-->TT) + (AG<-->AA)

Figure S3 Circos plot depicting the relationship between repetitive elements in the 12 current 0-1 scaffolds and the first draft assembly of 0-1 (Ellwood et al. 2010). (A) The outer-most ring represents the twelve 0-1 genomic scaffolds. Large numbers represent scaffold (chromosome) numbers ordered from largest (1) to smallest (12). Small numbers represent size in megabases. Alternating light and dark color patterns within each scaffold represent 0-1 scaffolded contigs (B) A heatmap depicting the density of repetitive elements along the twelve 0-1 scaffolds. Red represents higher repetitive content and green represents less repetitive content. (C) Coverage of the first draft genome assembly of P. teres f. teres isolate 0-1 (Ellwood et al. 2010) aligned to the 12 current 0-1 scaffolds. Dark blue represents regions of alignment and light blue represents regions of sequence absent in the first draft assembly.

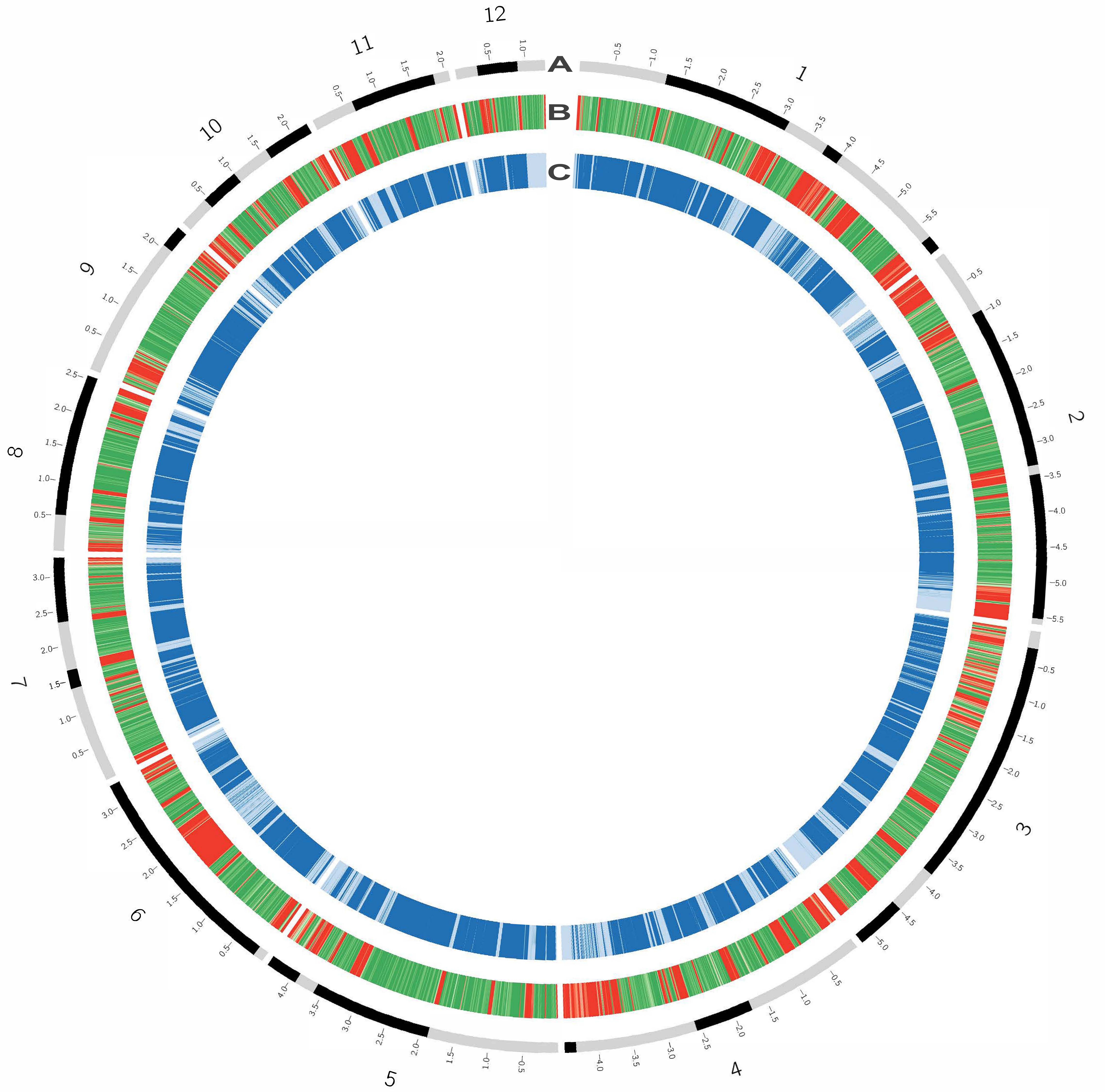
A
B
C
1
2
3
4
5
6
7
8
9
10
11
12

Table S1 Summary results of RepeatModeler annotated repetitive elements in the *P. teres* f. *teres* genome.

Total Sequences: 55

Total Length: 46,512,066 bp

Class	Count	bpMasked	%masked
=====	=====	========	=======
DNA	95	93014	0.20%
Academ	171	307448	0.66%
Academ-H	1	47	0.00%
CMC-EnSpm	9	549	0.00%
Dada	1	49	0.00%
MULE-MuDR	2	232	0.00%
P	1	78	0.00%
P-Fungi	3	194	0.00%
PIF-Harbinger	4	214	0.00%
TcMar-Fot1	4483	2504613	5.38%
TcMar-Mariner	1	32	0.00%
TcMar-Pogo	2	287	0.00%
TcMar-Sagan	1	47	0.00%
TcMar-Tc1	39	46880	0.10%
hAT-Ac	3	151	0.00%
hAT-Restless	359	389157	0.84%
LINE	--	--	--
Deceiver	1	75	0.00%
Tad1	181	238832	0.51%
LTR	2	135	0.00%
Copia	568	892466	1.92%
DIRS	1	43	0.00%
Gypsy	3016	4316731	9.28%
Ngaro	1	93	0.00%
RC	--	--	--
Helitron	3	259	0.00%
Retroposon	3	251	0.00%
Unknown	5948	3630402	7.81%
-	-------------	---------	----------
total interspersed	14899	12422279	26.71%
Low_complexity	783	38808	0.08%
Satellite	--	--	--
5S	3	190	0.00%
Simple_repeat	10021	2340408	5.03%
rRNA	29	46772	0.10%
-----------------------	-------------	---------	------------
Total	25735	14848457	31.92%

Table S2 Dinucleotide frequency calculations and RIP Indices identified in the 5 most numerous repeat families of the *P. teres* f. *teres* genome. TpA/ApT and (CpA+TpG)/(ApC+GpT) indices are calculated from frequencies of the 16 dinucleotide combinations possible. Evidence of RIP is defined as TpA/ApT > 2.0 and/or (CpA+TpG)/(ApC+GpT) < 0.7 (Galagar *et al.* 2003).

RepeatModeler Family	aa	ac	ag	at	ca	cc	cg	ct	ga	gc	gg	gt	ta	tc	tg	tt	TpA/ApT index[a]	(CpA+TpG)/(ApC+GpT)[a]
MOLLY_SN	6.9%	5.4%	9.7%	8.0%	2.6%	3.1%	4.8%	9.5%	4.7%	6.9%	3.1%	5.5%	15.6%	4.7%	2.6%	7.0%	1.97	0.48
GYPSY2	8.0%	5.3%	9.8%	10.0%	1.4%	2.4%	3.2%	10.0%	5.4%	3.7%	2.3%	5.3%	18.3%	5.6%	1.4%	8.1%	1.83	0.26
GYPSY3	7.1%	5.3%	9.0%	9.0%	2.2%	3.1%	3.9%	9.7%	5.6%	4.2%	3.0%	5.8%	15.5%	6.4%	2.6%	7.6%	1.72	0.44
GYPSY4	8.1%	4.9%	9.9%	10.9%	0.7%	1.9%	2.5%	10.5%	4.9%	3.2%	1.9%	5.2%	20.2%	5.5%	0.8%	8.7%	1.85	0.15
LMR1	9.0%	4.6%	10.4%	10.6%	1.0%	1.4%	2.1%	10.6%	4.5%	4.2%	1.4%	4.7%	20.1%	4.9%	1.0%	9.6%	1.89	0.21
0-1 genomic DNA	6.2%	5.6%	8.4%	7.7%	4.2%	3.8%	5.1%	8.8%	5.7%	6.6%	3.8%	5.6%	11.8%	5.9%	4.3%	6.6%	1.53	0.76

[a] According to Galagan et al. (2003), TpA/ApT > 2.0 or CpA+TpG /ApC+GpT < 0.7 corresponds to regions predicted as RIP-degenerated in Neurospora crassa
